# Supplementary material for: Evaluation of Quinazolin-2,4-Dione Derivatives as Promising Antibacterial Agents: Synthesis, In Vitro, In Silico ADMET and Molecular Docking Approaches
Source: Molecules. 2024 Nov 22;29(23):5529. doi: 10.3390/molecules29235529 (PMC11643521; doi:10.3390/molecules29235529)
Supplement: Supplementary file 1 [file molecules-29-05529-s001.zip › molecules-3251295-supplementary.pdf]

SUPPLEMENTARY FILE

Evaluation of quinazolin-2,4-dione Derivatives as Promising Antibacterial Agents: Synthesis, *In vitro*, *In silico* ADMET and Molecular Docking Approaches

Figure S1: IR spectrum of compound 1... 1

Figure S2: ^{1}H-NMR spectrum of compound 1... 2

Figure S3: ^{13}C-NMR spectrum of compound 1... 3

Figure S4: Mass spectrum of compound 1.. 4

Figure S5: IR spectrum of compound 2a... 5

Figure S6: ^{1}H-NMR spectrum of compound 2a... 6

Figure S7: ^{13}C-NMR spectrum of compound 2a... 7

Figure S8: Mass spectrum of compound 2a... 8

Figure S9: IR spectrum of compound 2b. 9

Figure S10: ^{1}H-NMR spectrum of compound 2b. 10

Figure S11: ^{13}C-NMR spectrum of compound 2b 11

Figure S12: Mass spectrum of compound 2b..12

Figure S13: IR spectrum of compound 2c... 13

Figure S14: ^{1}H-NMR spectrum of compound 2c... 14

Figure S15: ^{13}C-NMR spectrum of compound 2c 15

Figure S16: Mass spectrum of compound 2c... 16

Figure S17: IR spectrum of compound 3a 17

Figure S18: ^{1}H-NMR spectrum of compound 3a. 18

Figure S19: ^{13}C-NMR spectrum of compound 3a 19

Figure S20: Mass spectrum of compound 3a... 20

Figure S21: IR spectrum of compound 3b.. 21

Figure S22: ^{1}H-NMR spectrum of compound 3b.. 22

Figure S23: ^{13}C-NMR spectrum of compound 3b 23

Figure S24: Mass spectrum of compound 3b. 24

Figure S25: IR spectrum of compound 3c. 25

Figure S26: ^{1}H-NMR spectrum of compound 3c.. 26

Figure S27: ^{13}C-NMR spectrum of compound 3c 27
Figure S28: Mass spectrum of compound 3c.. 28
Figure S29: IR spectrum of compound 3d. 29
Figure S30: ^{1}H-NMR spectrum of compound 3d.. 30
Figure S31: ^{13}C-NMR spectrum of compound 3d 31
Figure S32: Mass spectrum of compound 3d.. 32
Figure S33: IR spectrum of compound 4a. 33
Figure S34: ^{1}H-NMR spectrum of compound 4a.. 34
Figure S35: ^{13}C-NMR spectrum of compound 4a 35
Figure S36: Mass spectrum of compound 4a... 36
Figure S37: IR spectrum of compound 4b. 37
Figure S38: ^{1}H-NMR spectrum of compound 4b.. 38
Figure S39: ^{13}C-NMR spectrum of compound 4b 39
Figure S40: Mass spectrum of compound 4b.. 40
Figure S41: IR spectrum of compound 4c. 41
Figure S42: ^{1}H-NMR spectrum of compound 4c.. 42
Figure S43: Mass spectrum of compound 4c... 43
Figure S44: IR spectrum of compound 4d. 44
Figure S45: ^{1}H-NMR spectrum of compound 4d.. 45
Figure S46: Mass spectrum of compound 4d... 46
Figure S47. 3D interactions of the other docked compounds against 2W9S……………..53
Figure S48. 3D interactions of the other docked compounds against 1Q46……………..54

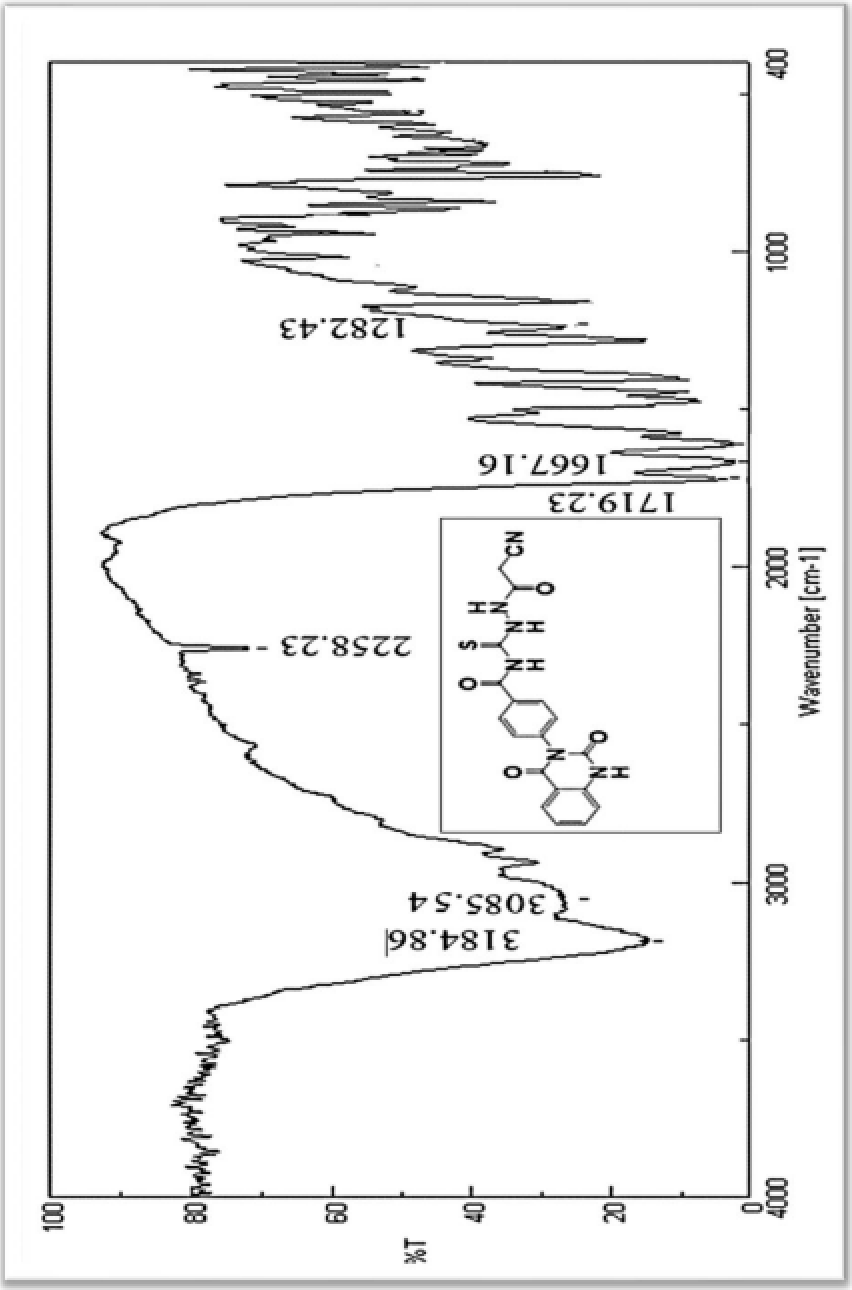

Figure S1: IR spectrum of compound 1

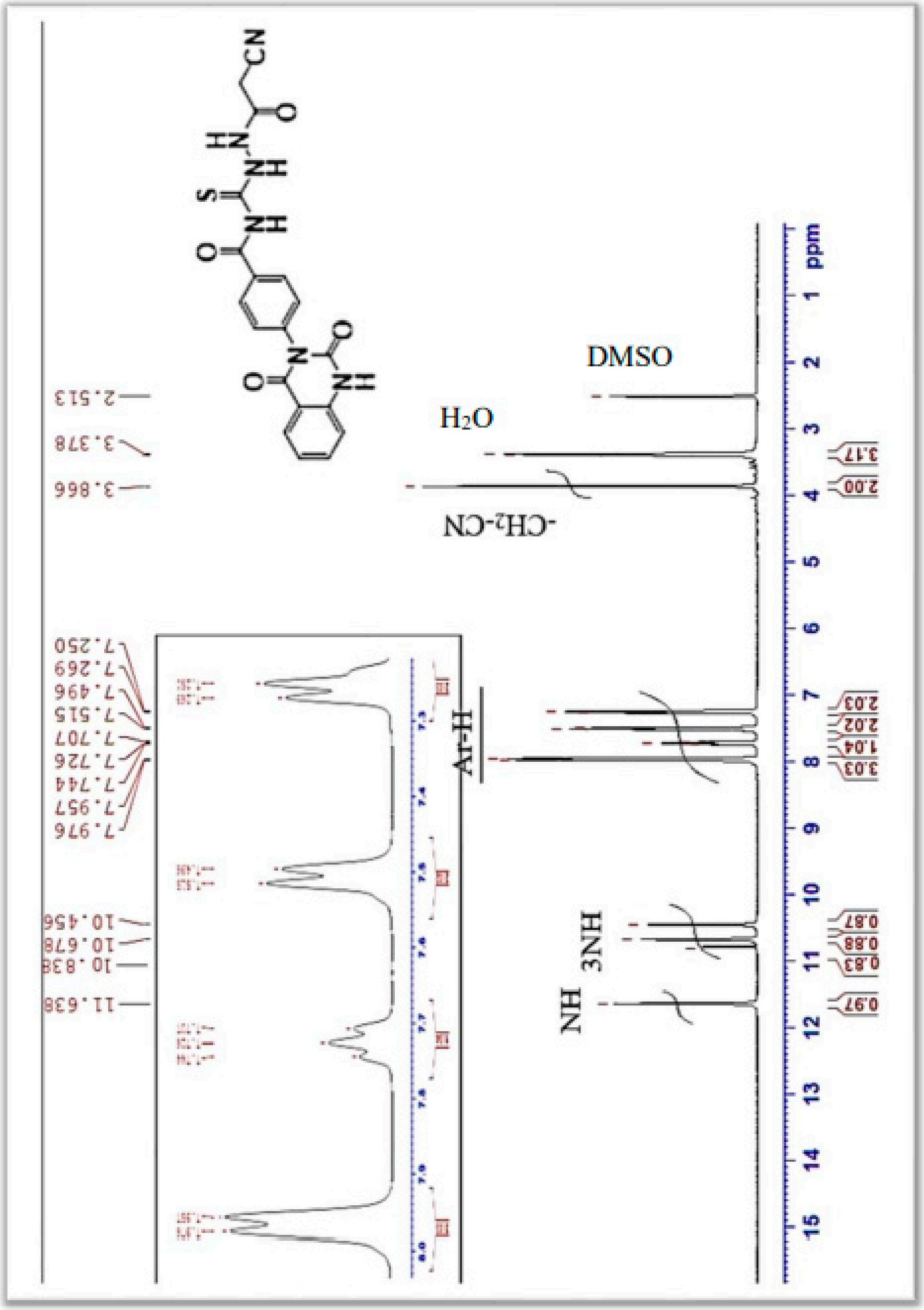

Figure S2: ^{1}H-NMR spectrum of compound 1

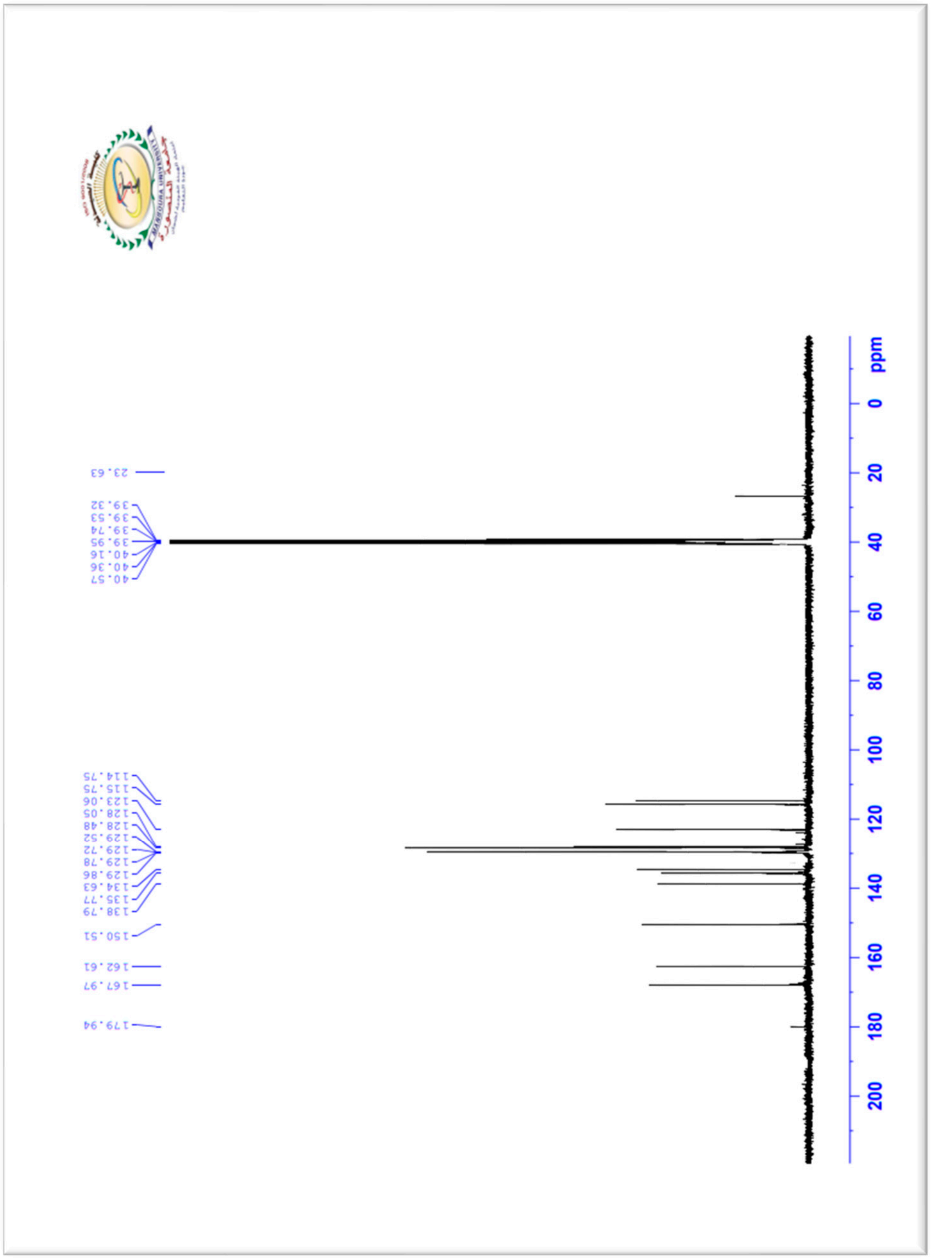

Figure S3: - ^{13}C-NMR analysis for compound 1

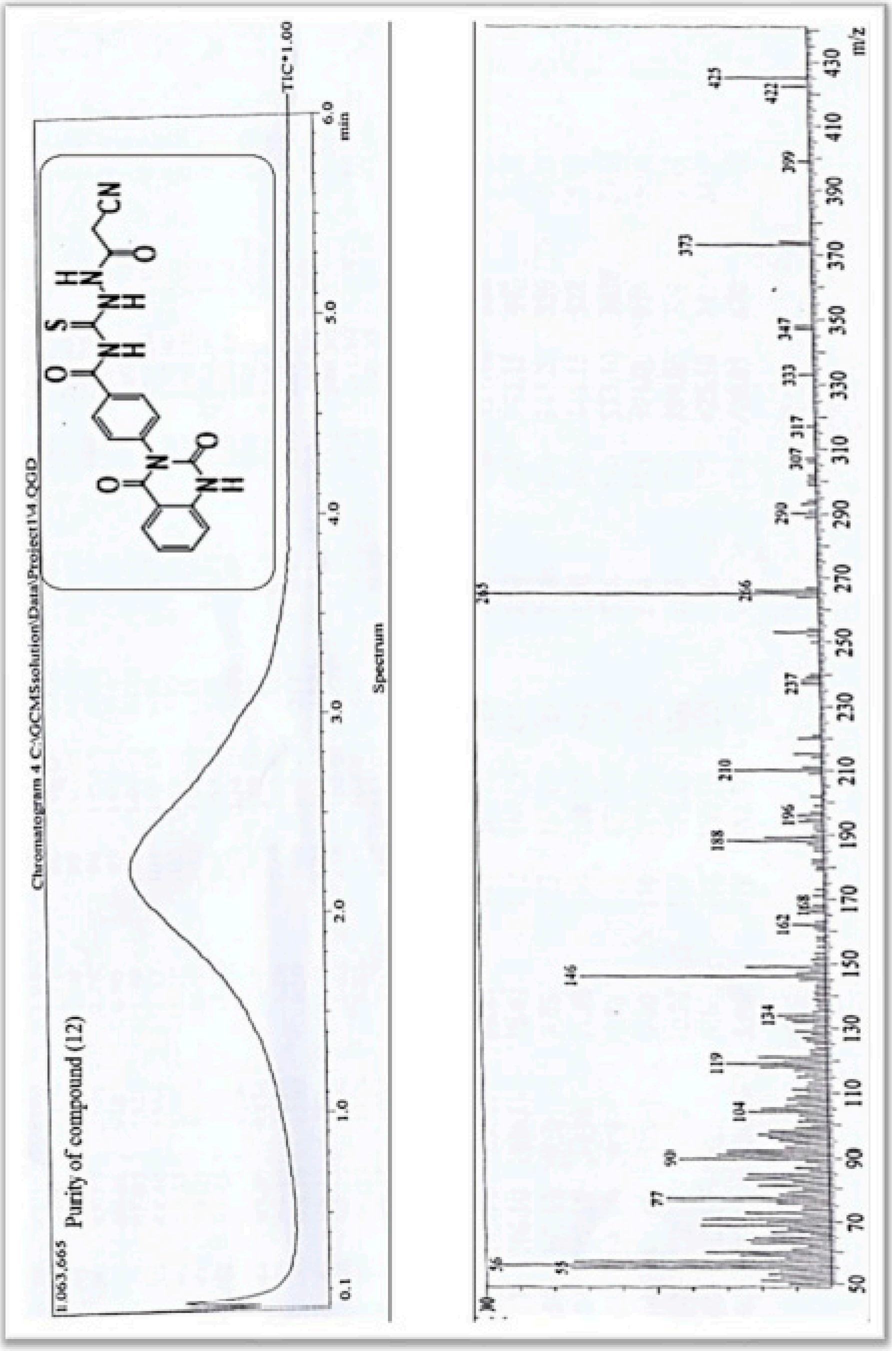

Figure S4: Mass spectrum of compound 1

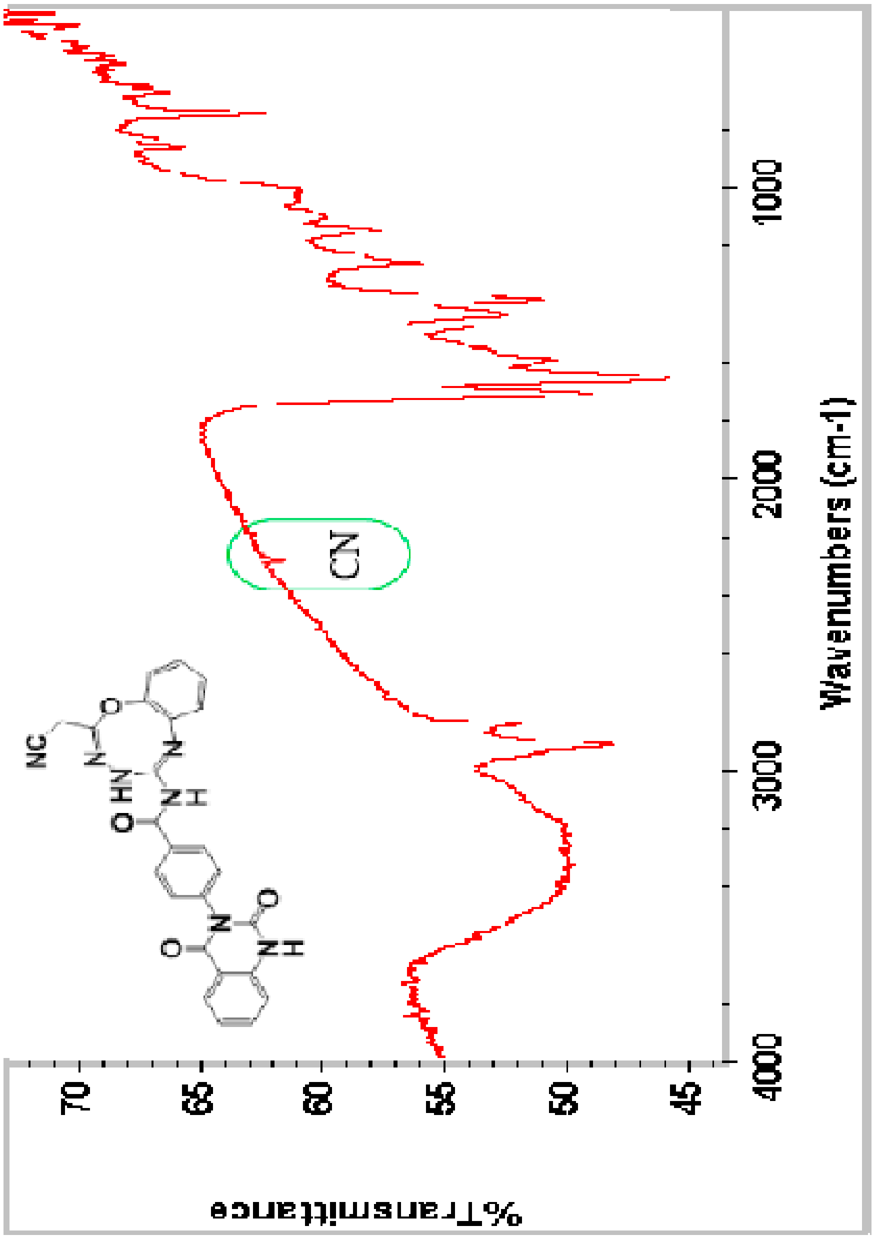

Figure S5: IR spectrum of compound 2a

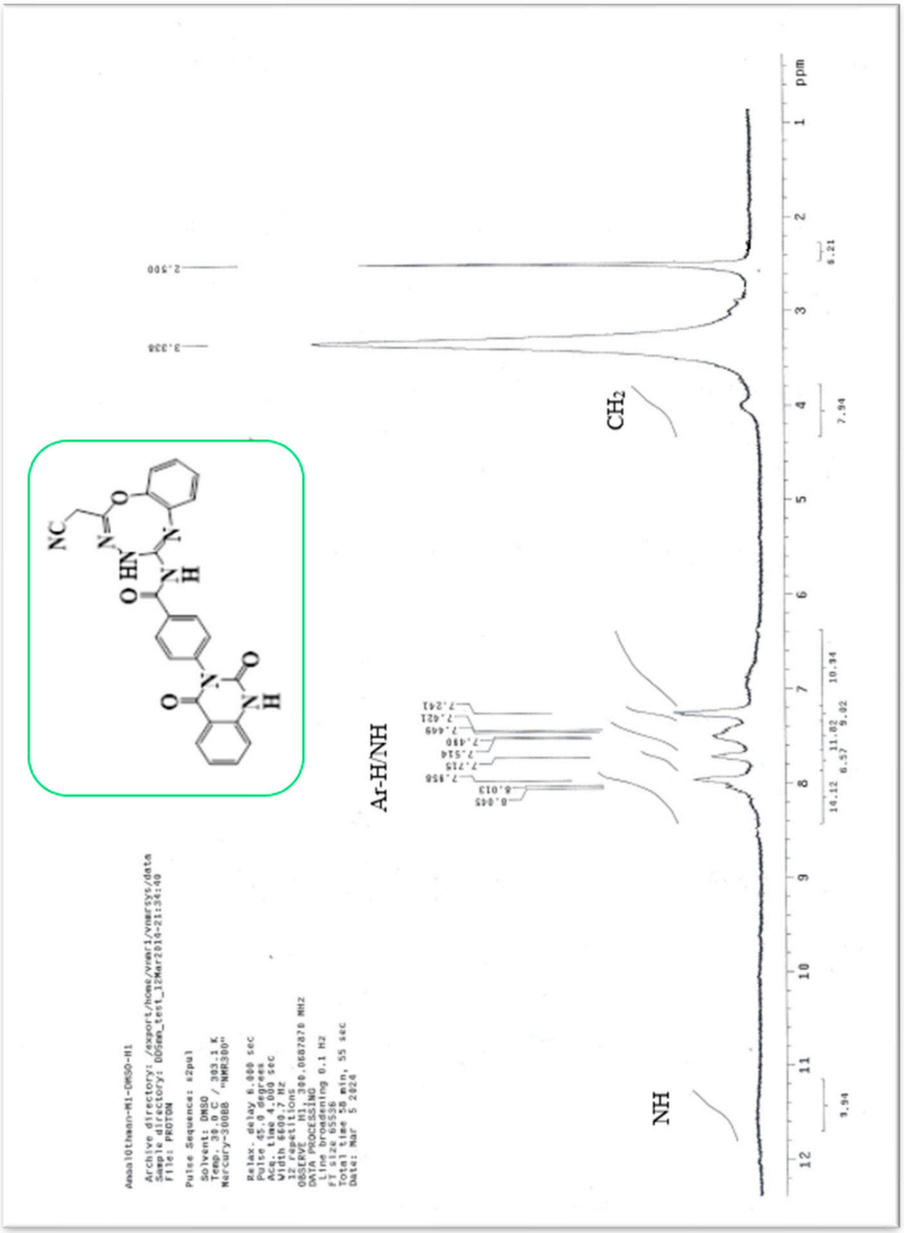

Figure S6: ¹H-NMR spectrum of compound 2a

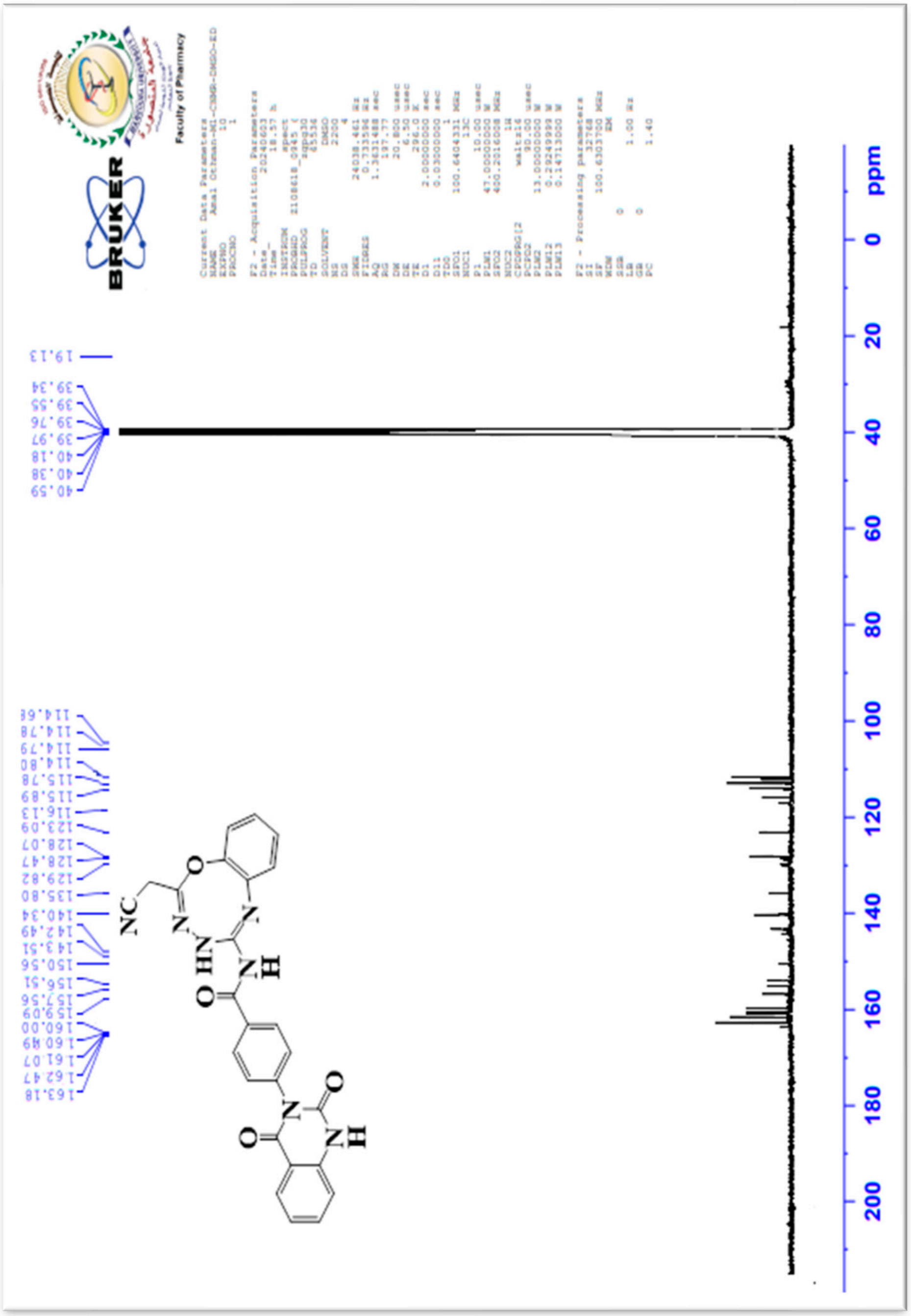

Figure S7: ^{13}C-NMR spectrum of compound 2a

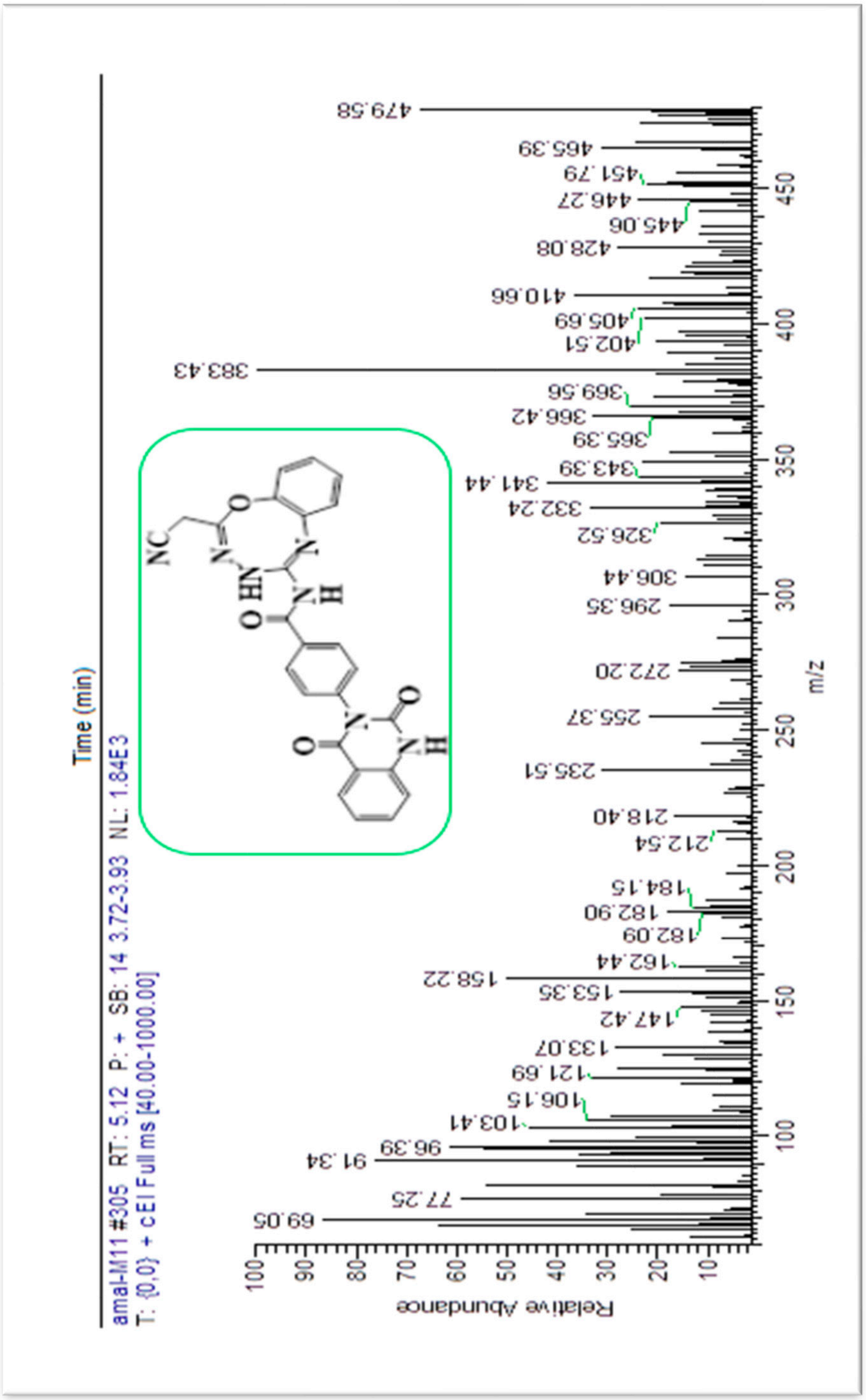

Figure S8: Mass spectrum of compound 2a

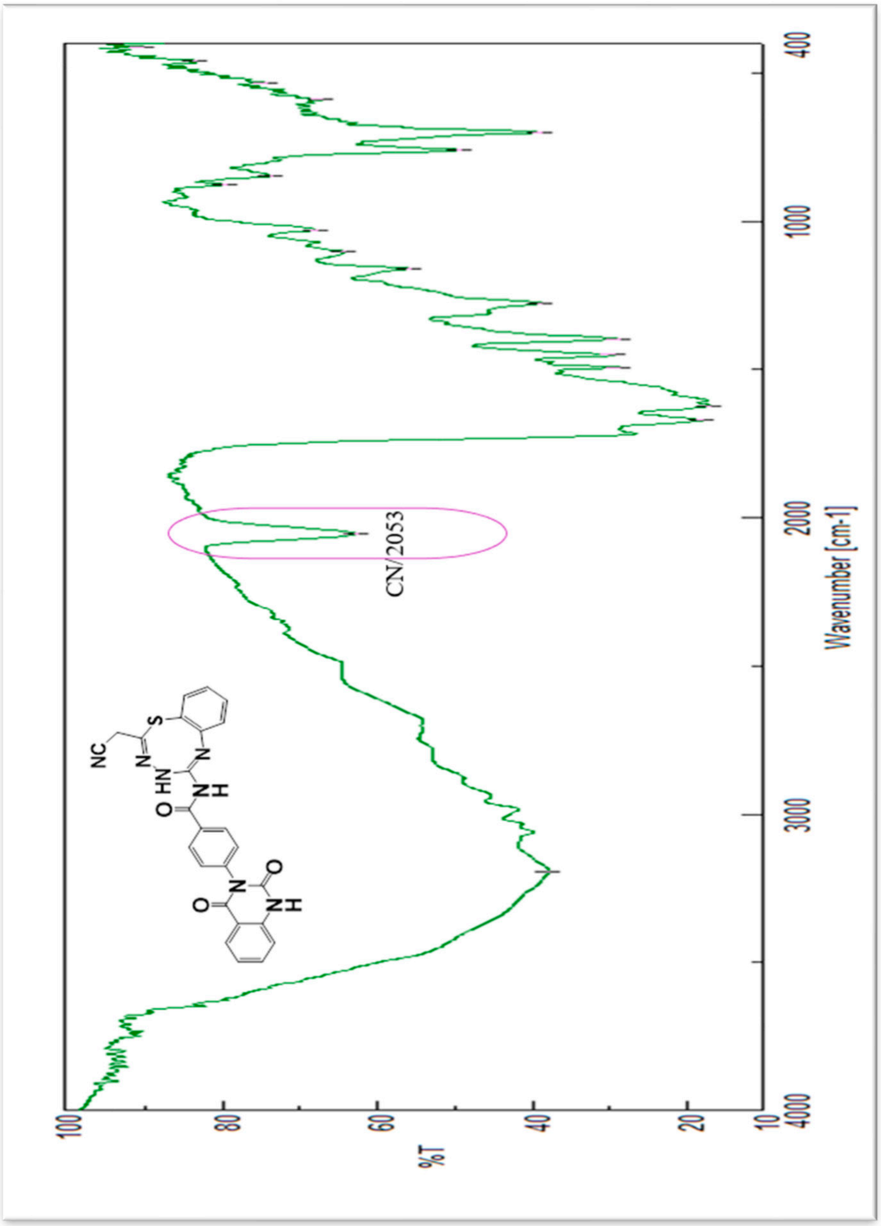

Figure S9: IR spectrum of compound 2b

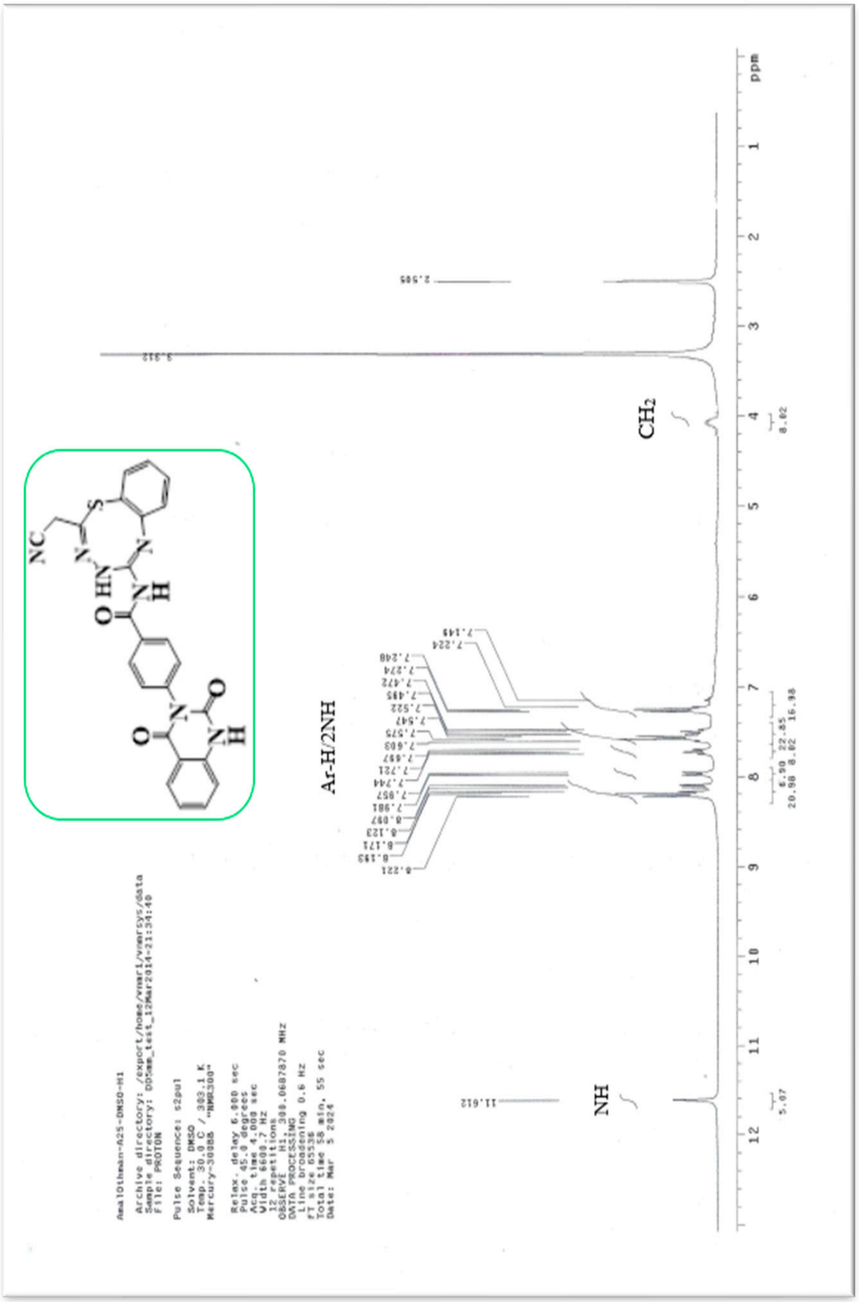

Figure S10: ^{1}H-NMR spectrum of compound 2b

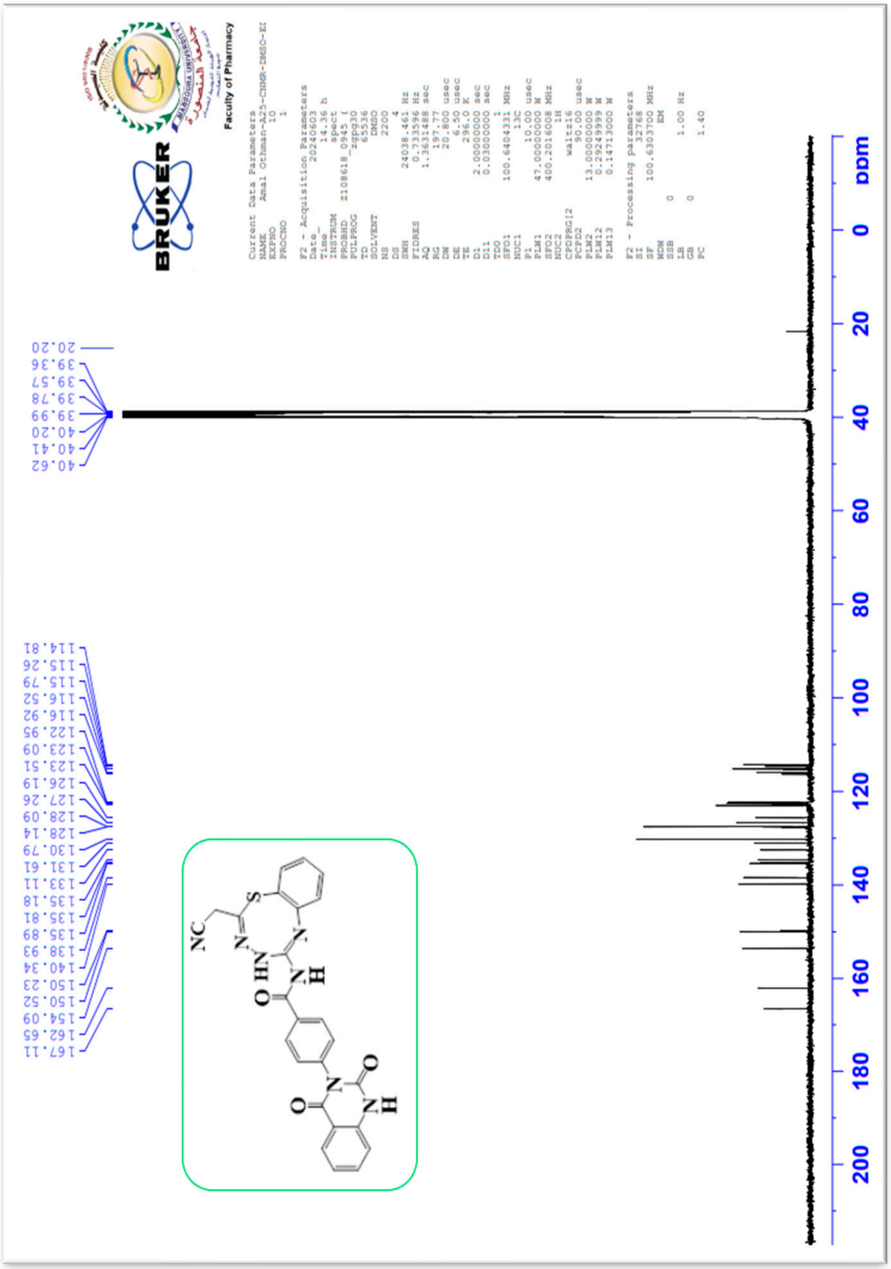

Figure S11: ^{1}H-NMR spectrum of compound 2b

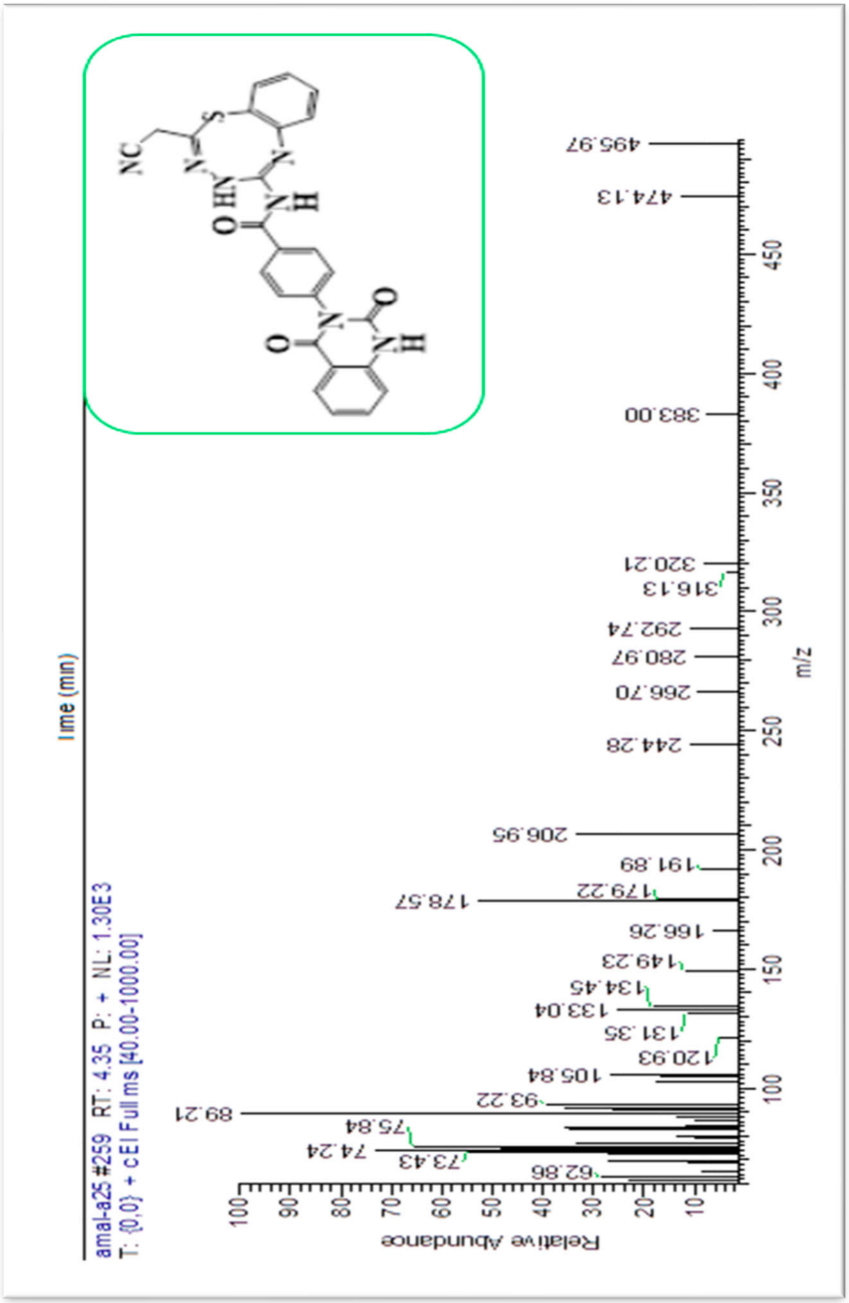

Figure S12: Mass spectrum of compound 2b

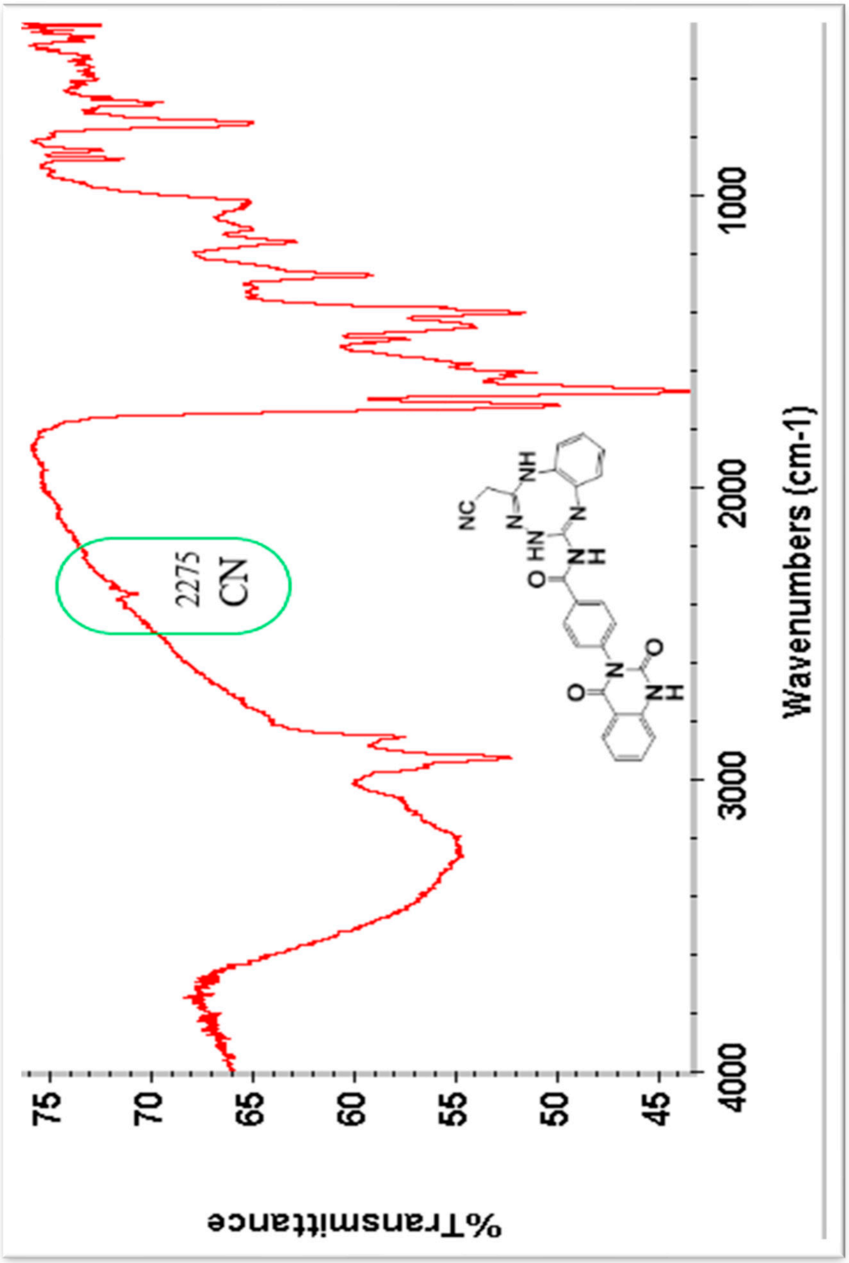

Figure S13: IR spectrum of compound 2c

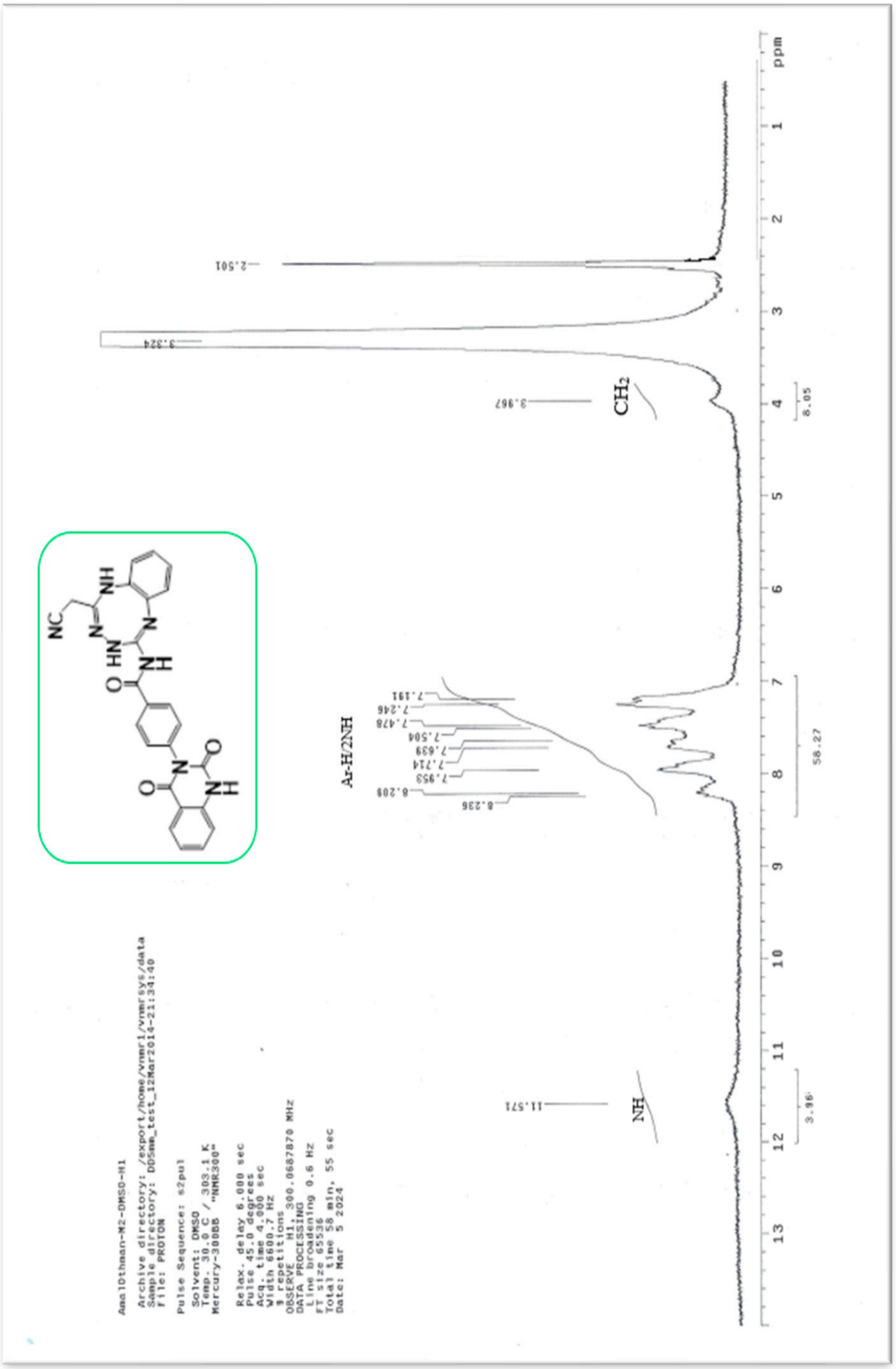

Figure S14: ^{1}H-NMR spectrum of compound 2c

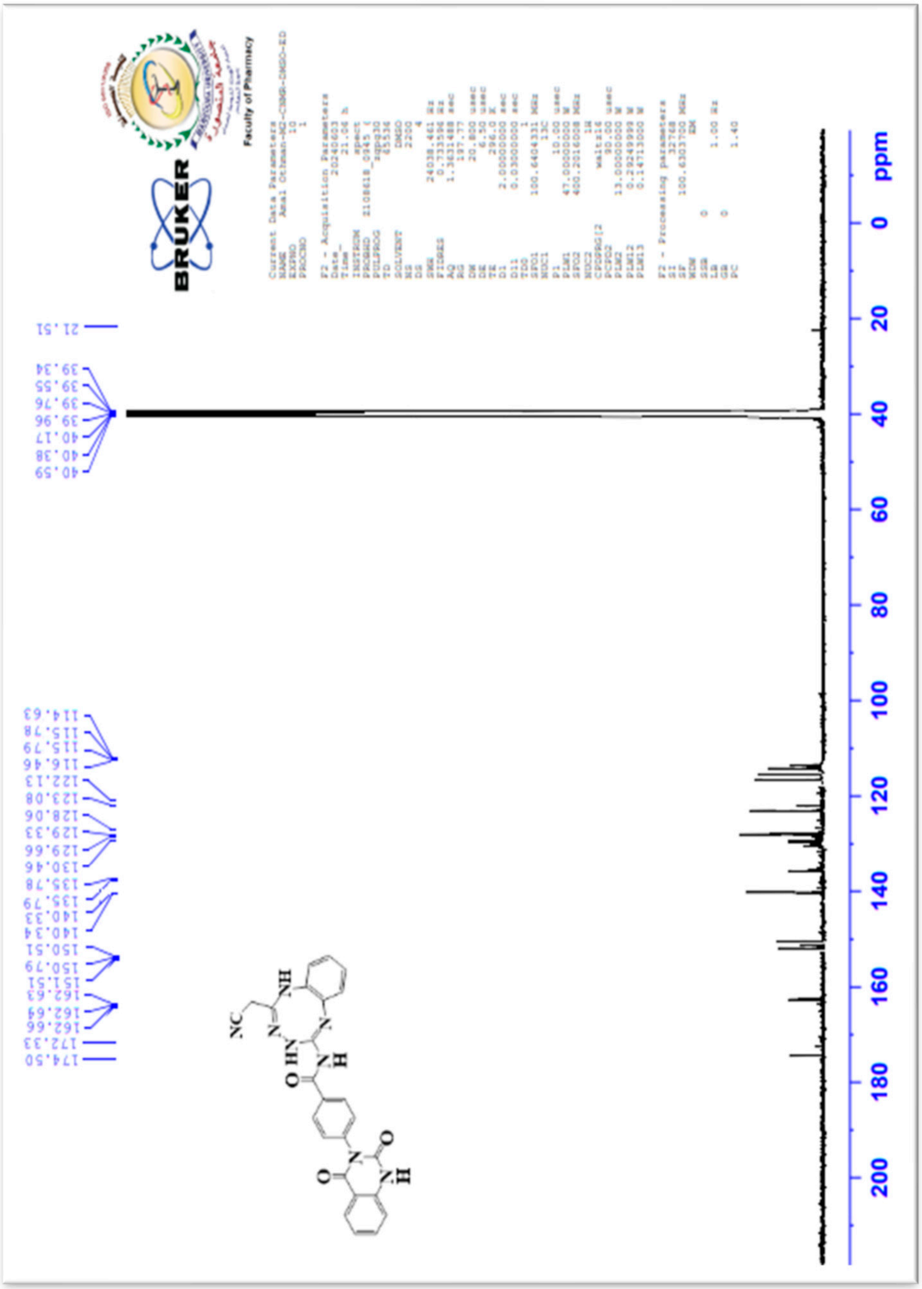

Figure S15: ^{13}C-NMR spectrum of compound 2c

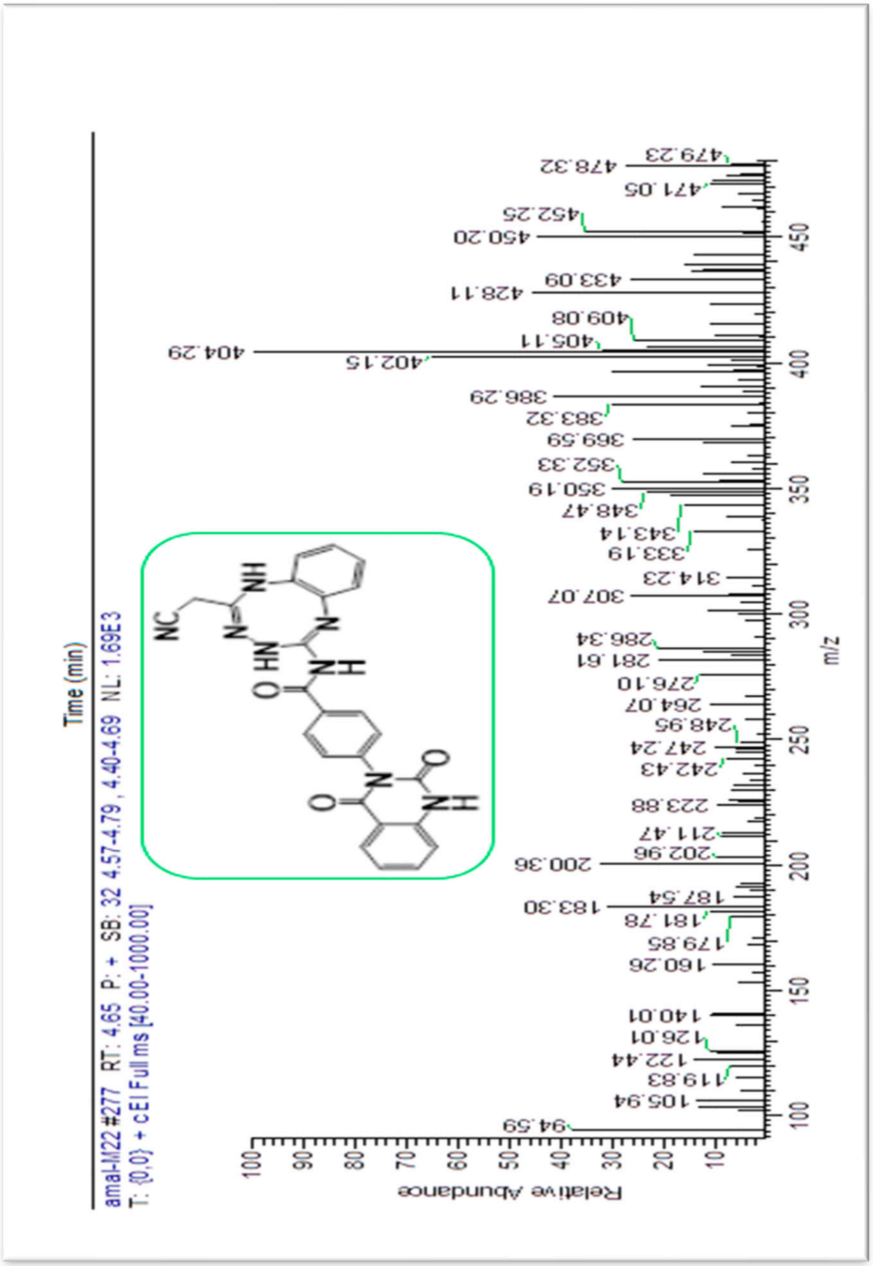

Figure S16: Mass spectrum of compound 2c

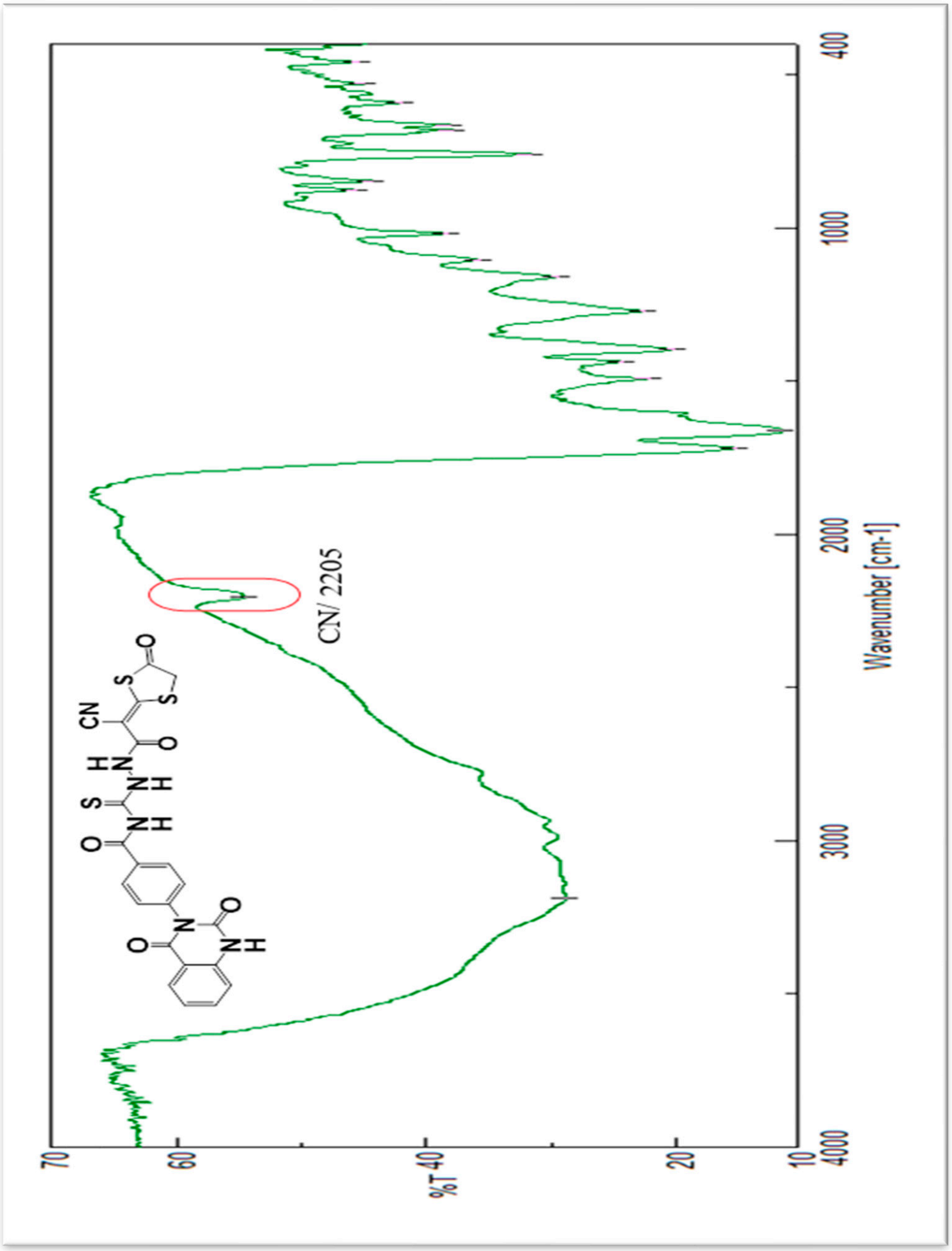

Figure S17: IR spectrum of compound 3a

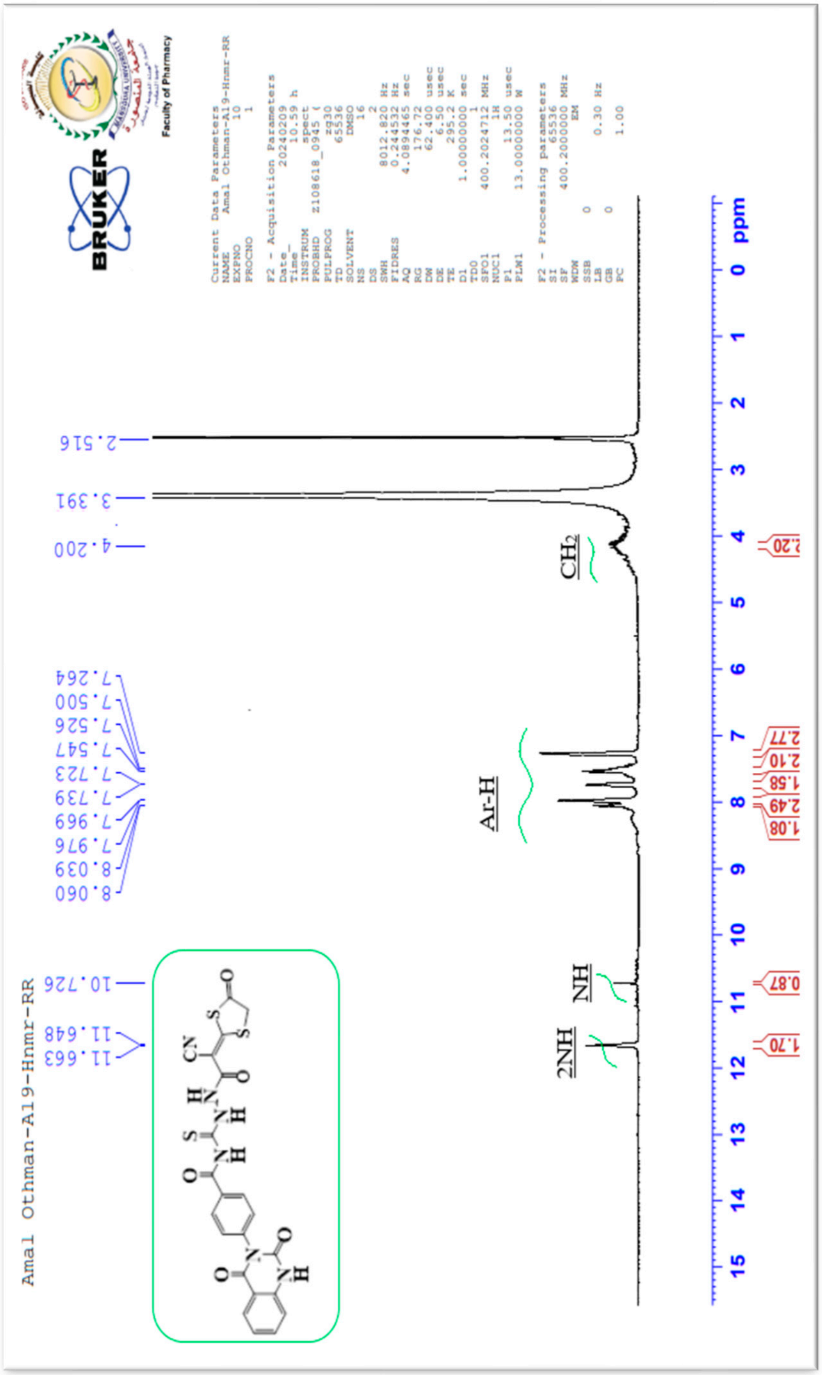

Figure S18: ^{1}H-NMR spectrum of compound 3a

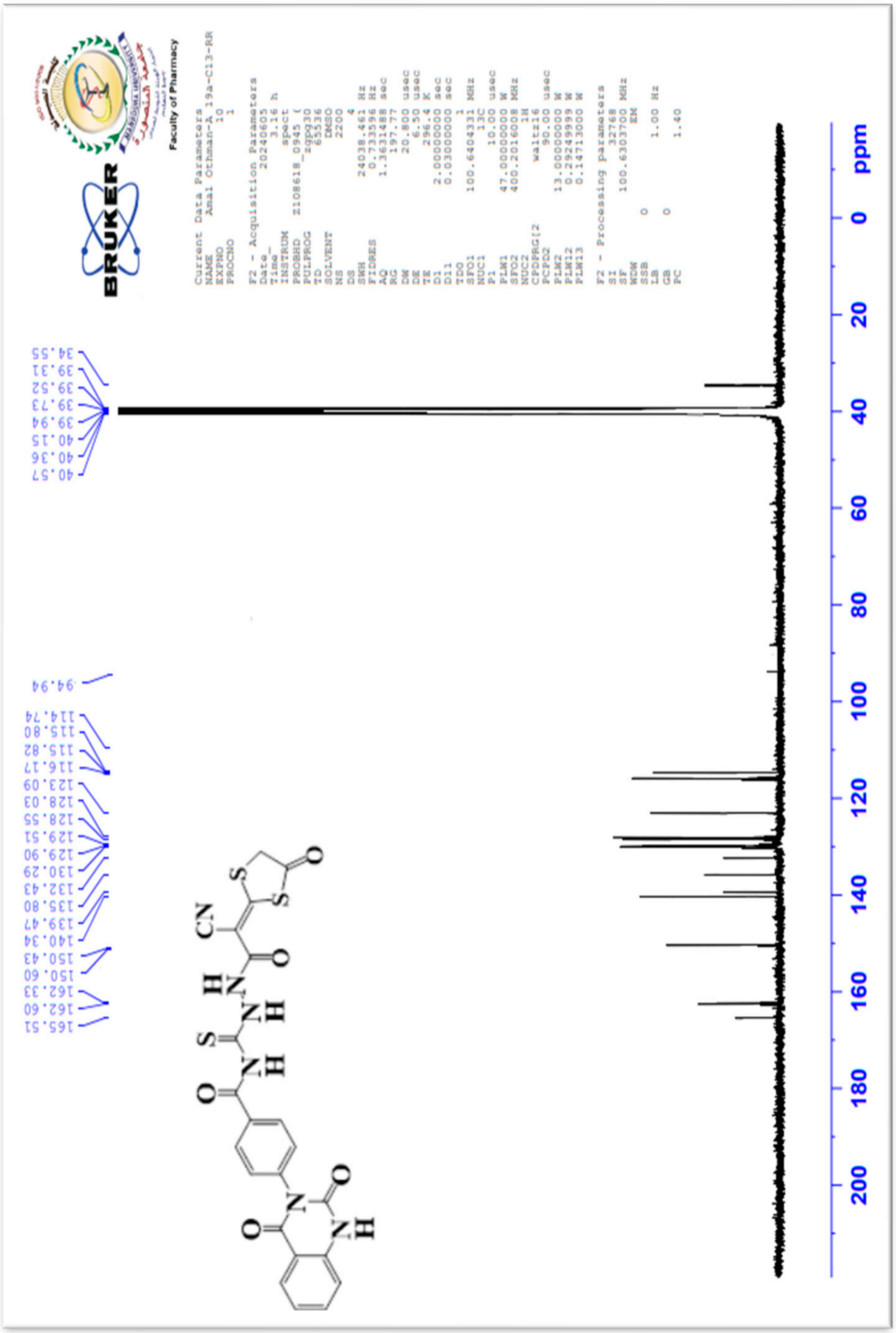

Figure S19: ^{13}C-NMR spectrum of compound 3a

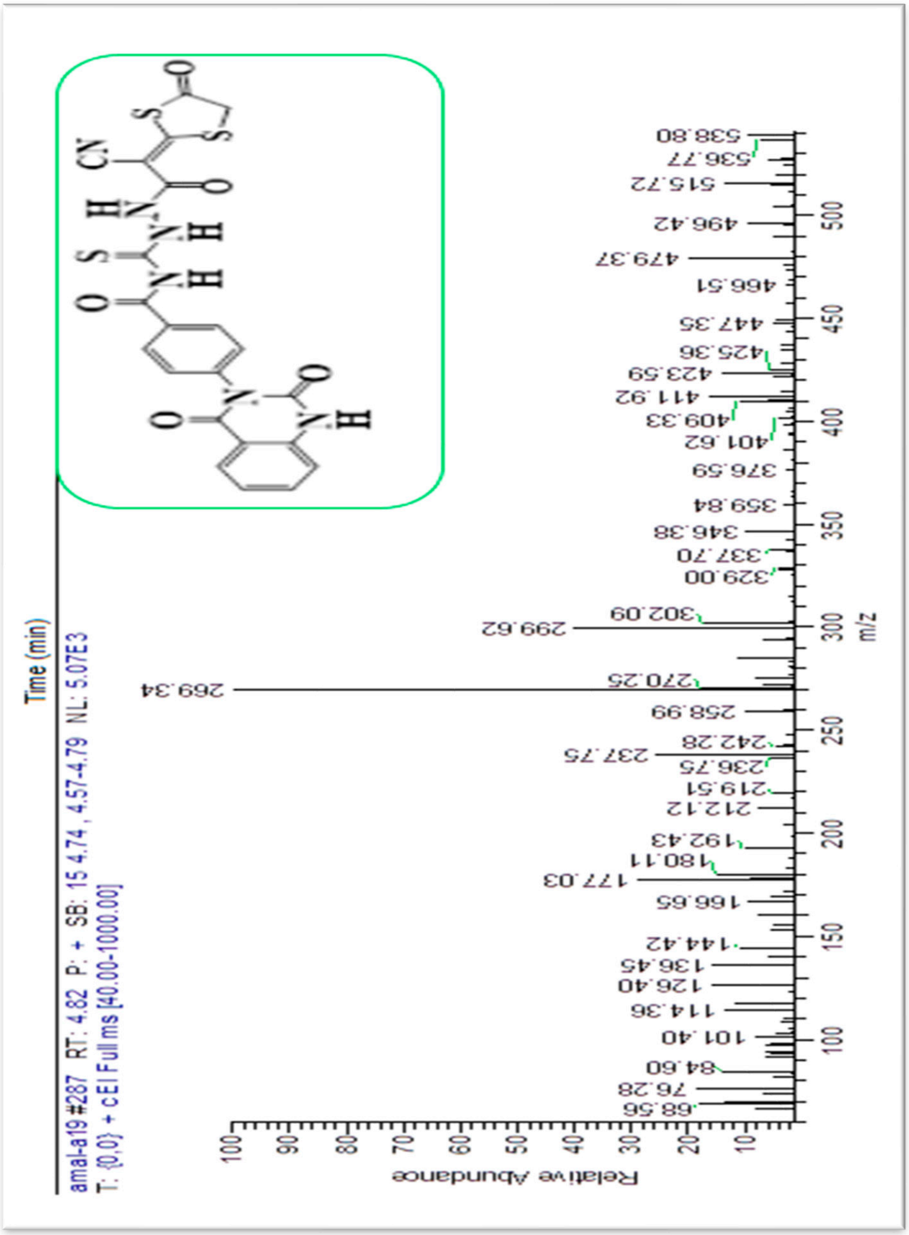

Figure S20: Mass spectrum of compound 3a

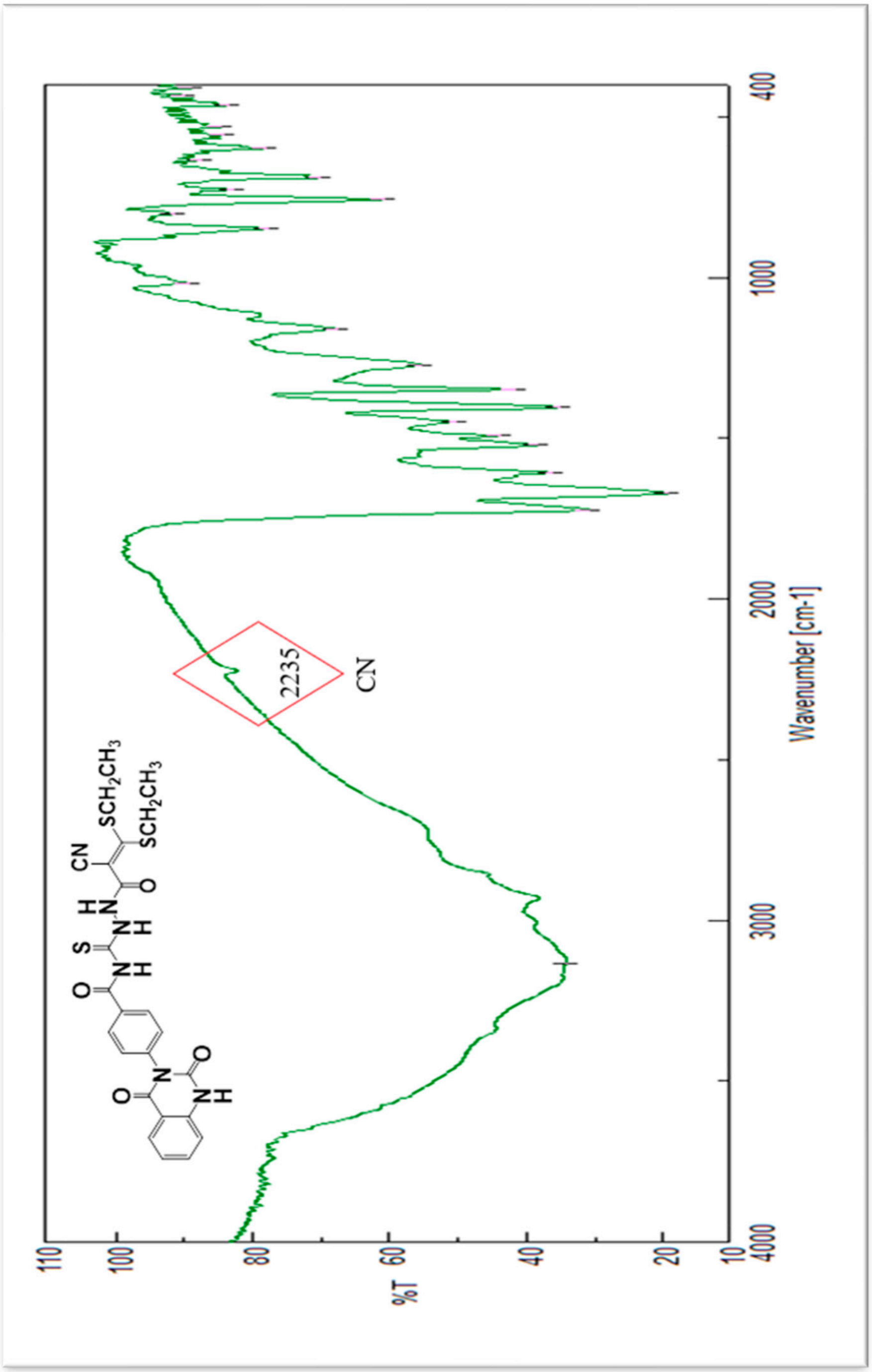

Figure S21: IR spectrum of compound 3b

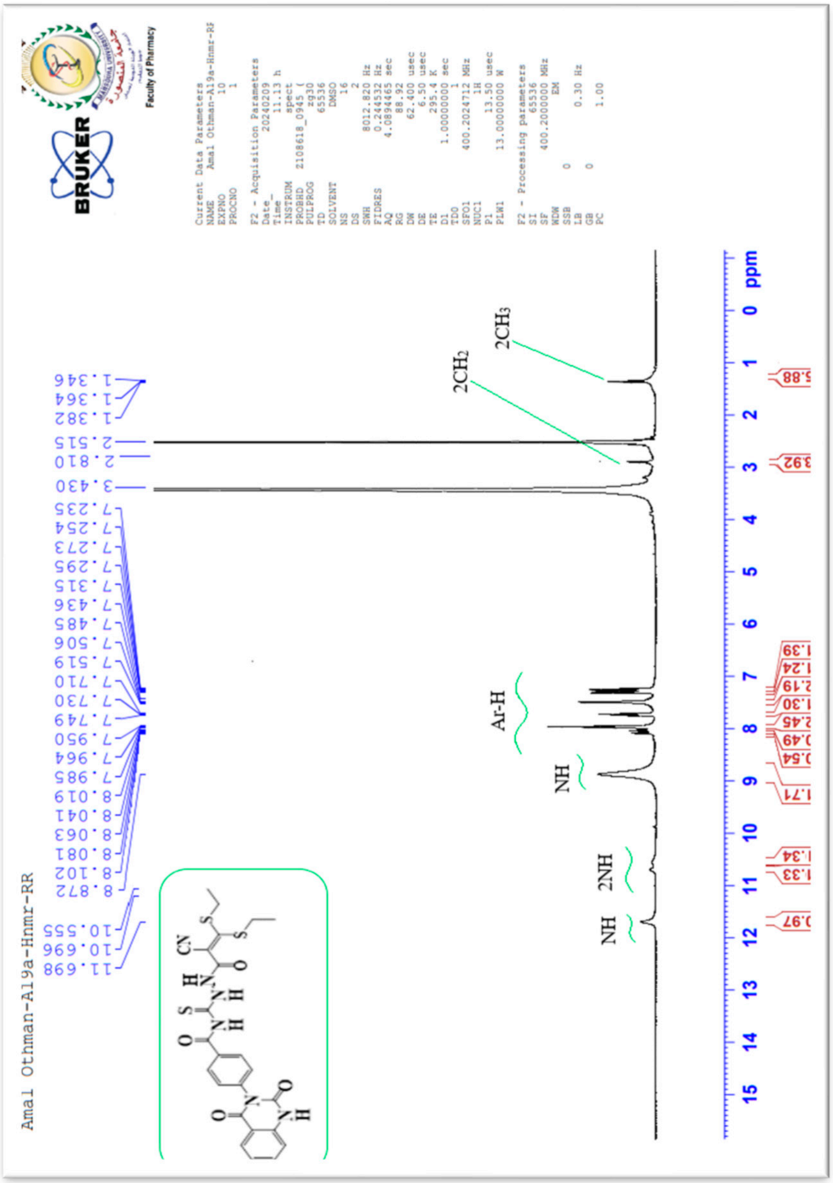

Figure S22: ^{1}H-NMR spectrum of compound 3b

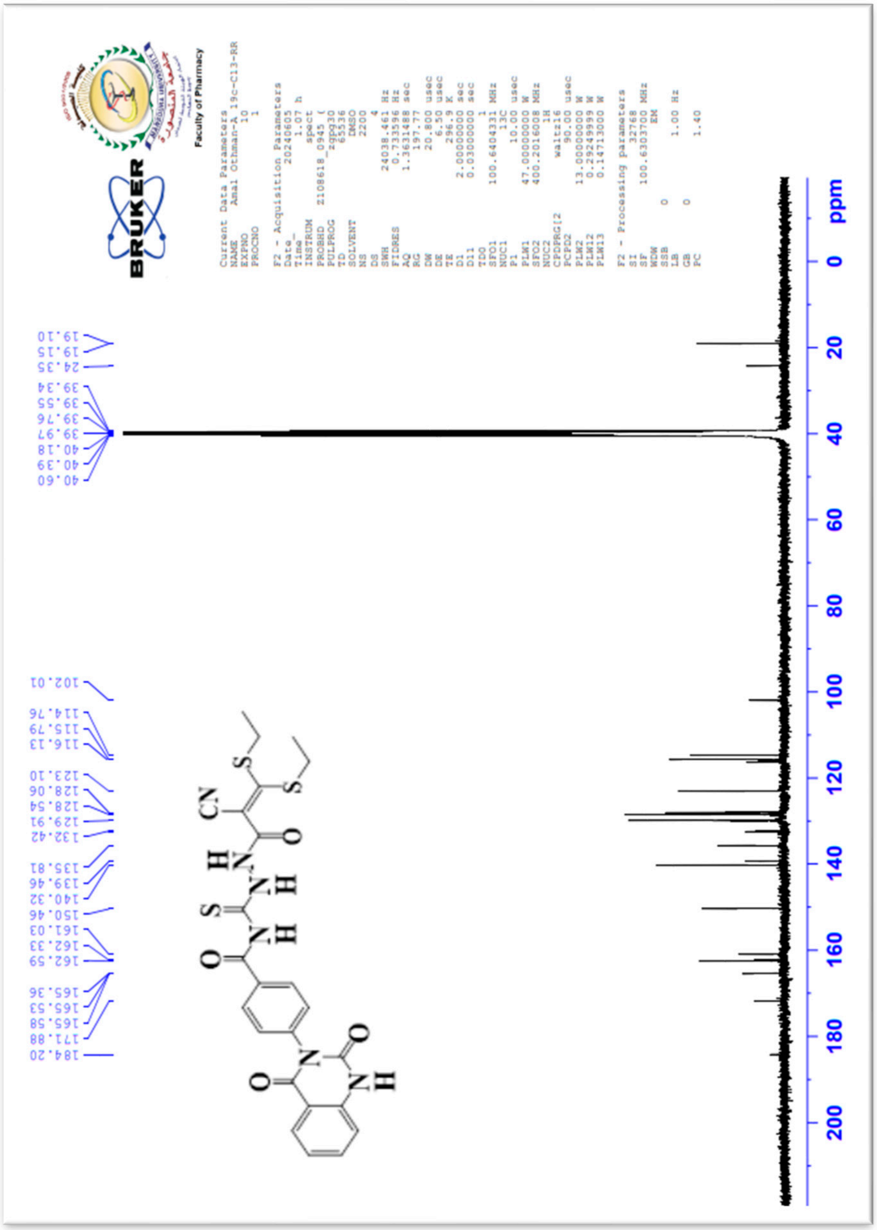

Figure S23: ^{13}C-NMR spectrum of compound 3b

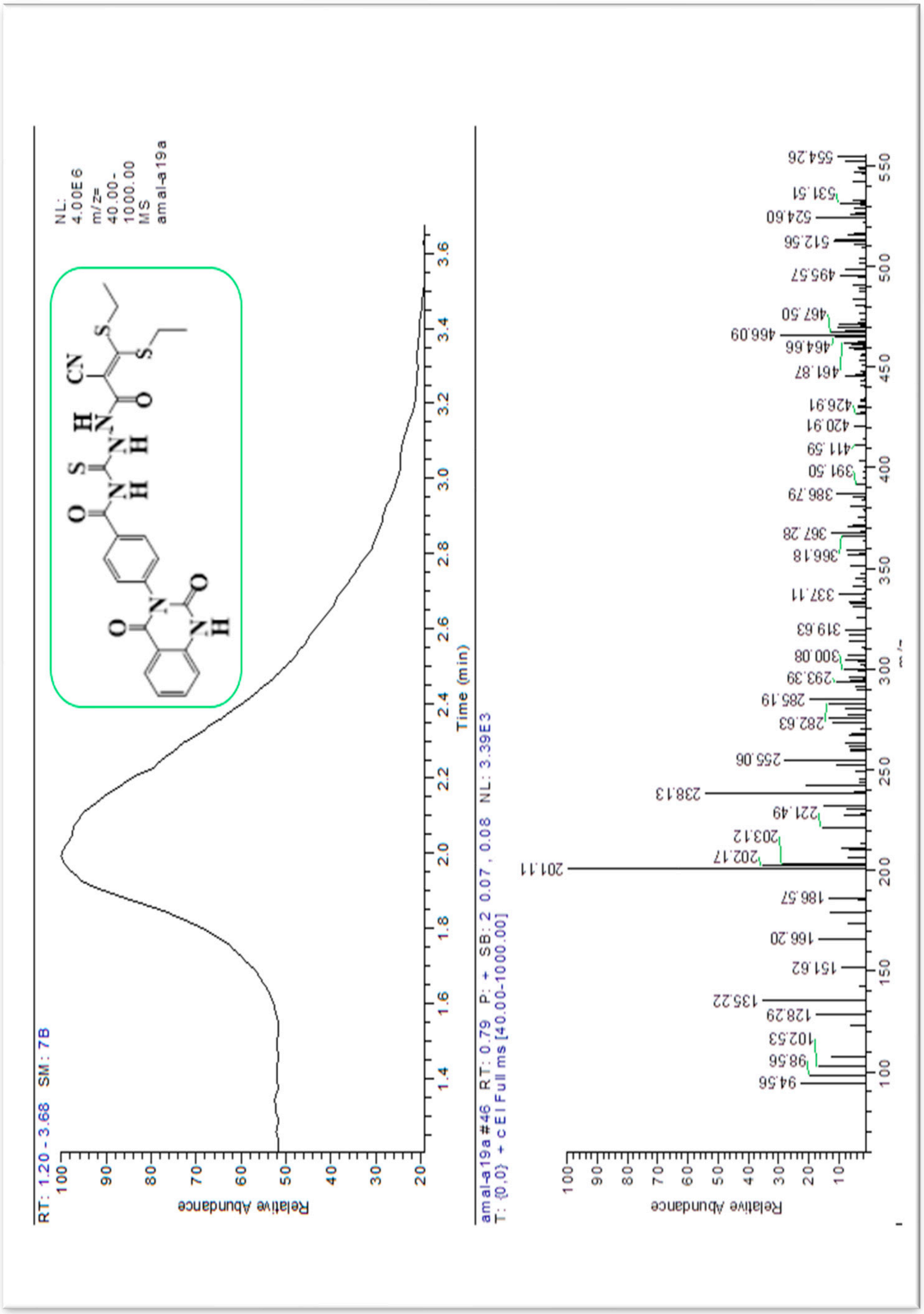

Figure S24: Mass spectrum of compound 3b

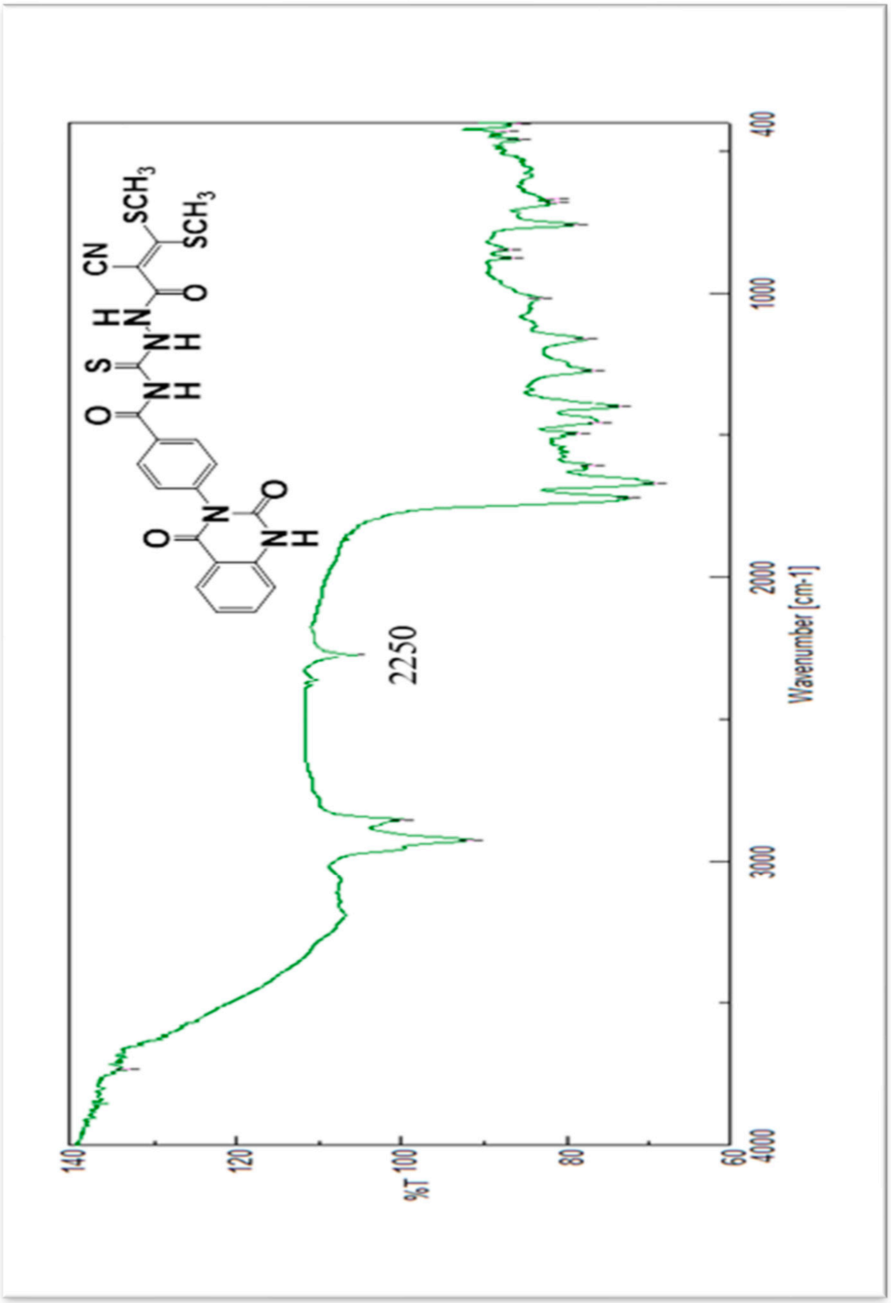

Figure S25: IR spectrum of compound 3c

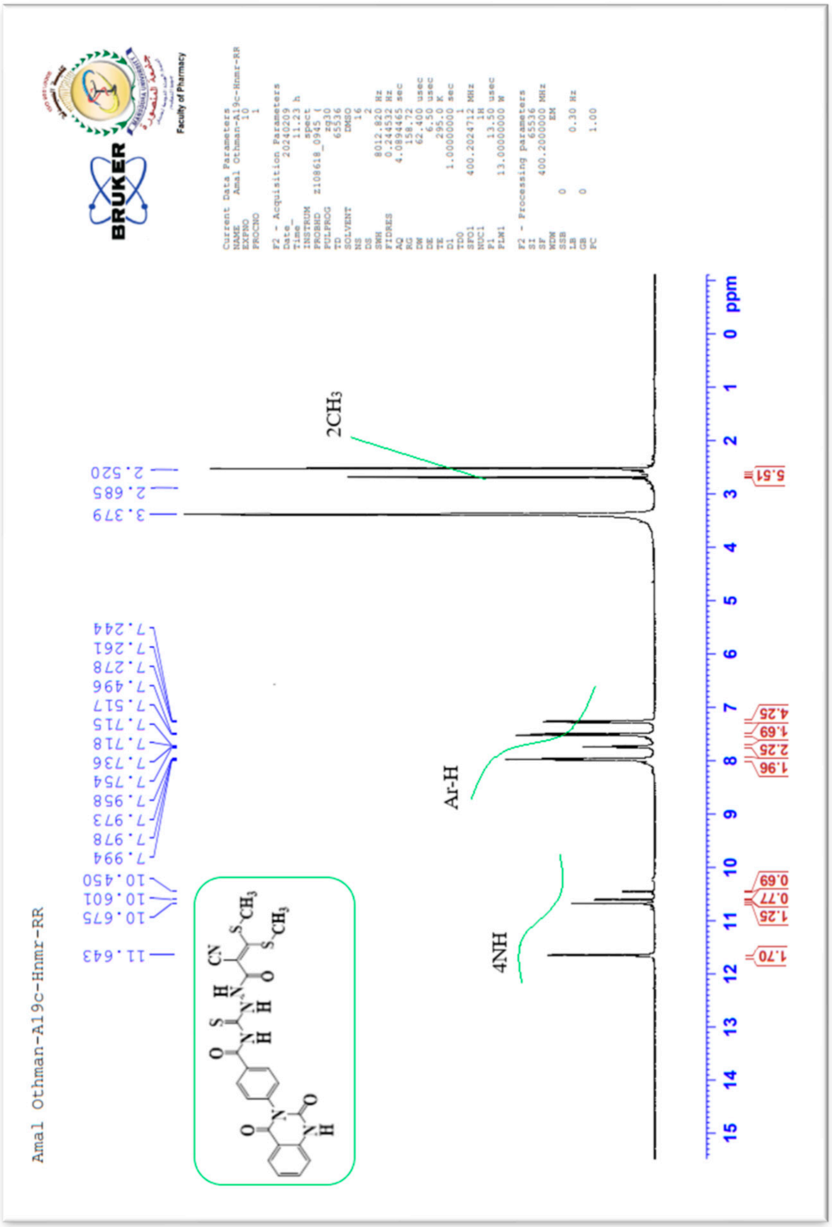

Figure S26: ^{1}H-NMR spectrum of compound 3c

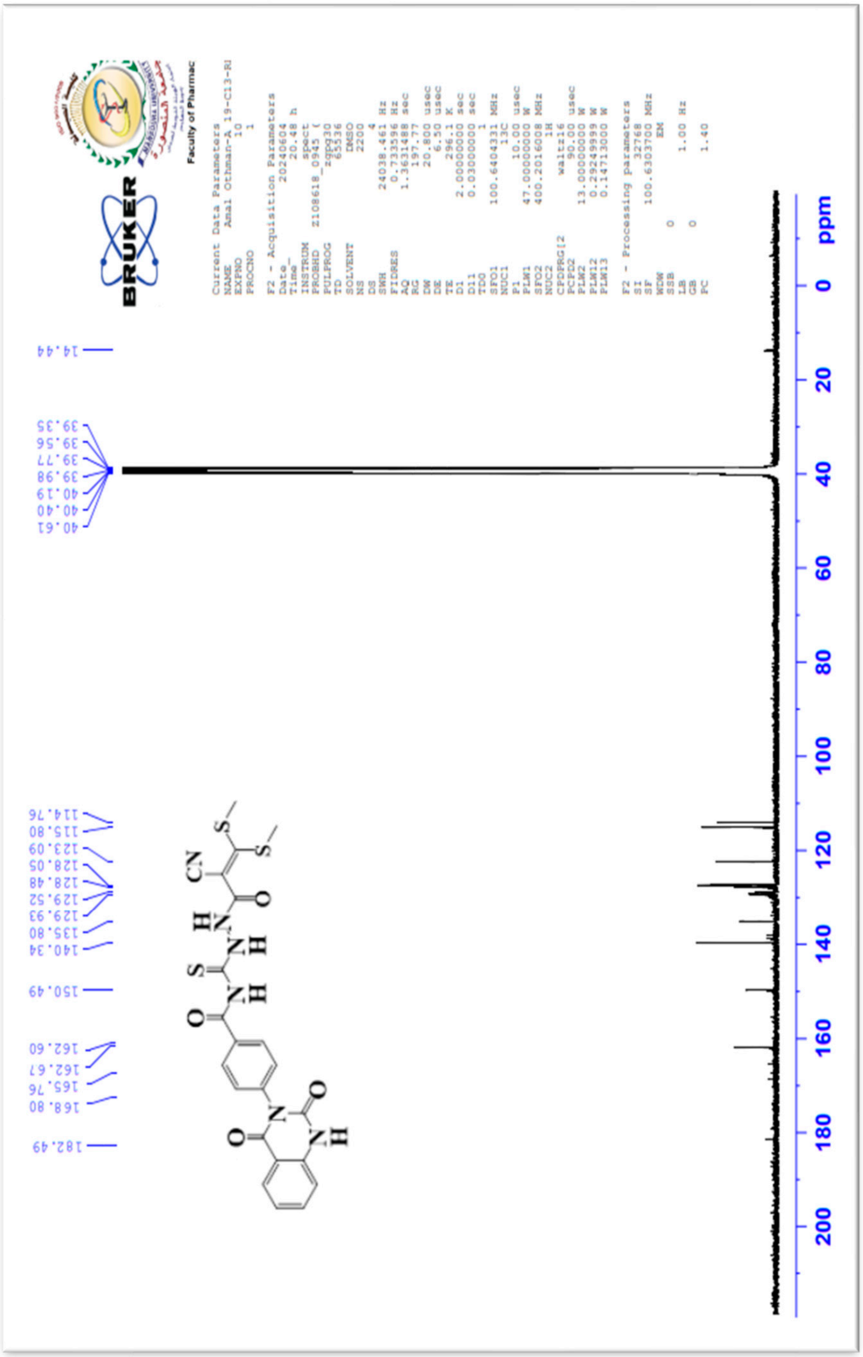

Figure S27: ^{13}C-NMR spectrum of compound 3c

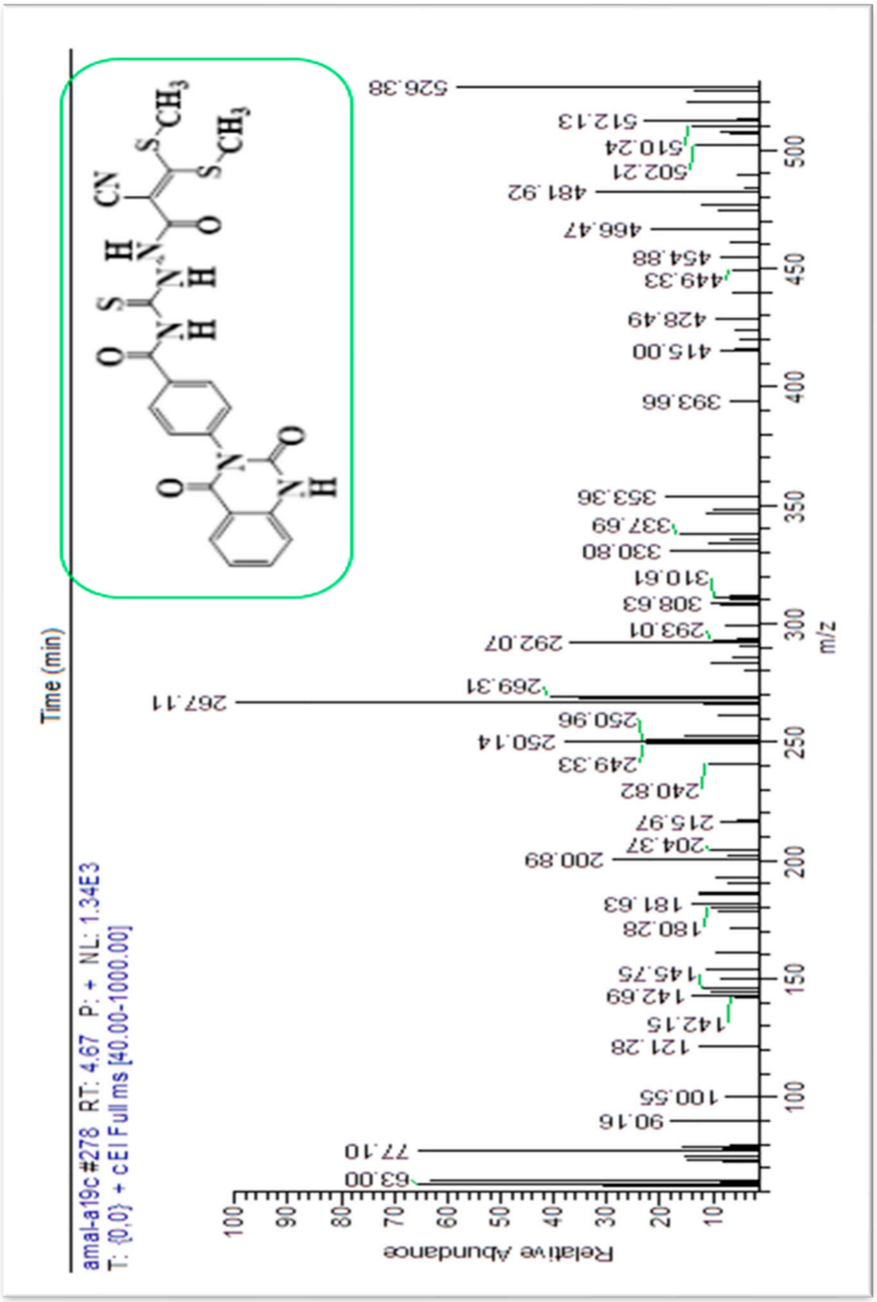

Figure 28: Mass spectrum of compound 3c

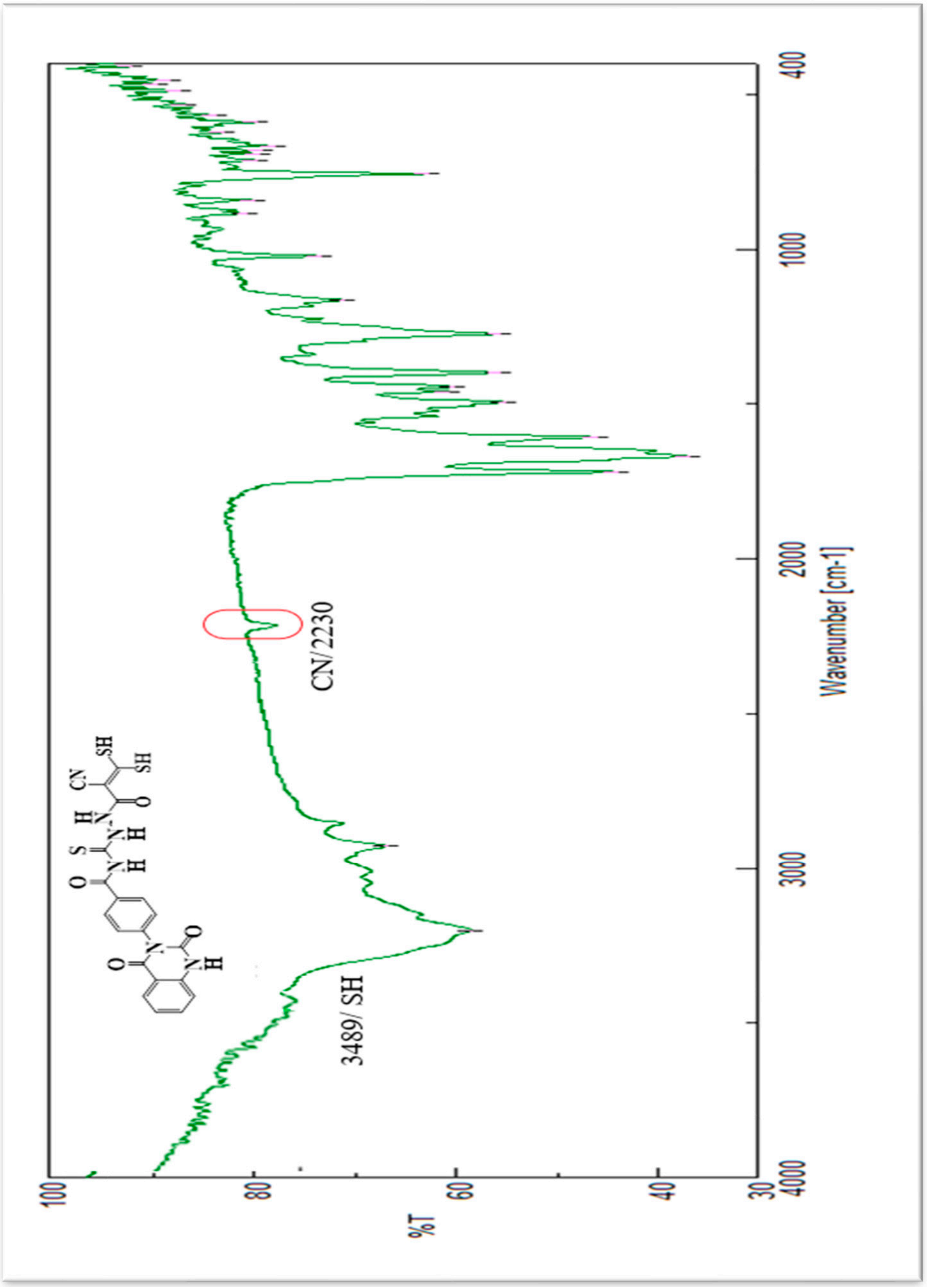

Figure S29: IR spectrum of compound 3d

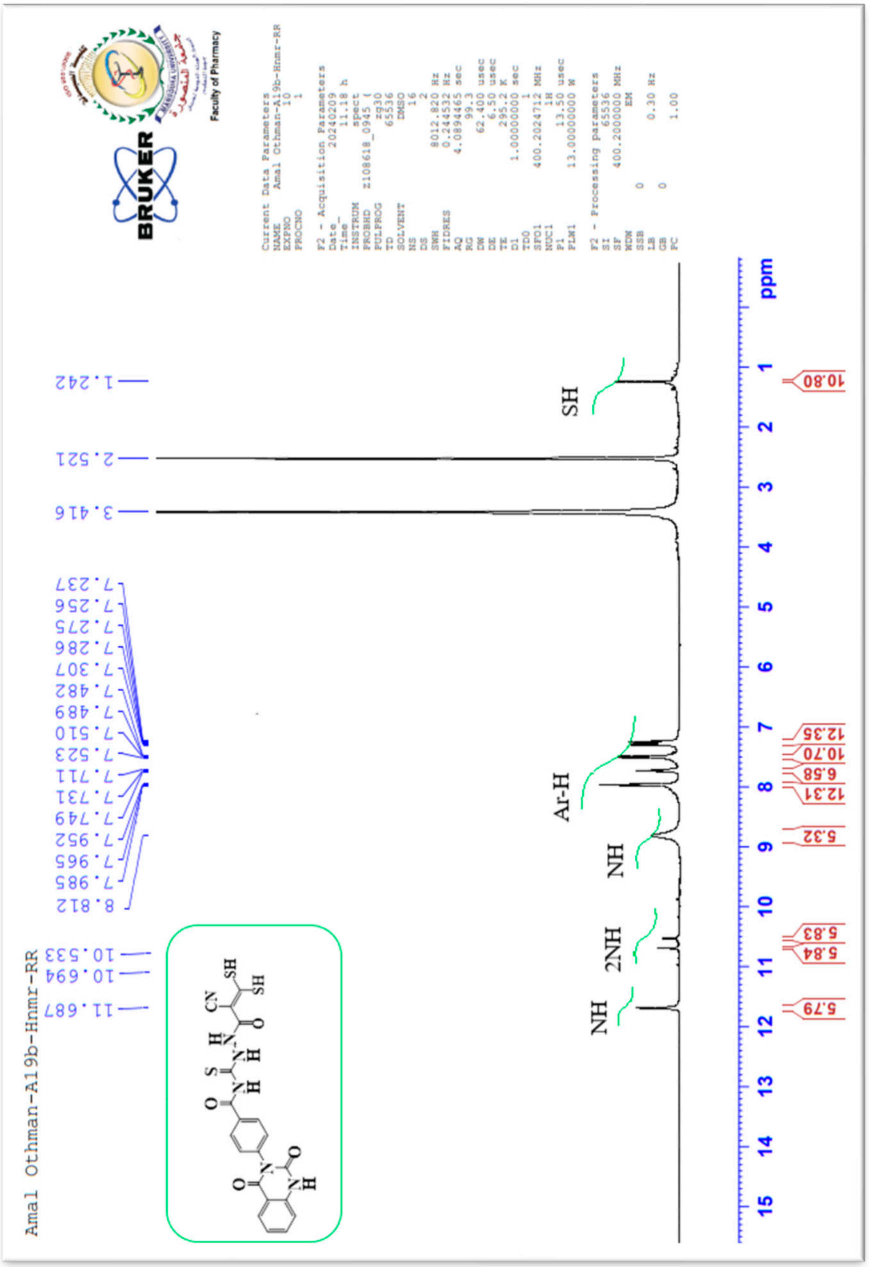

Figure S30: ^{1}H-NMR spectrum of compound 3d

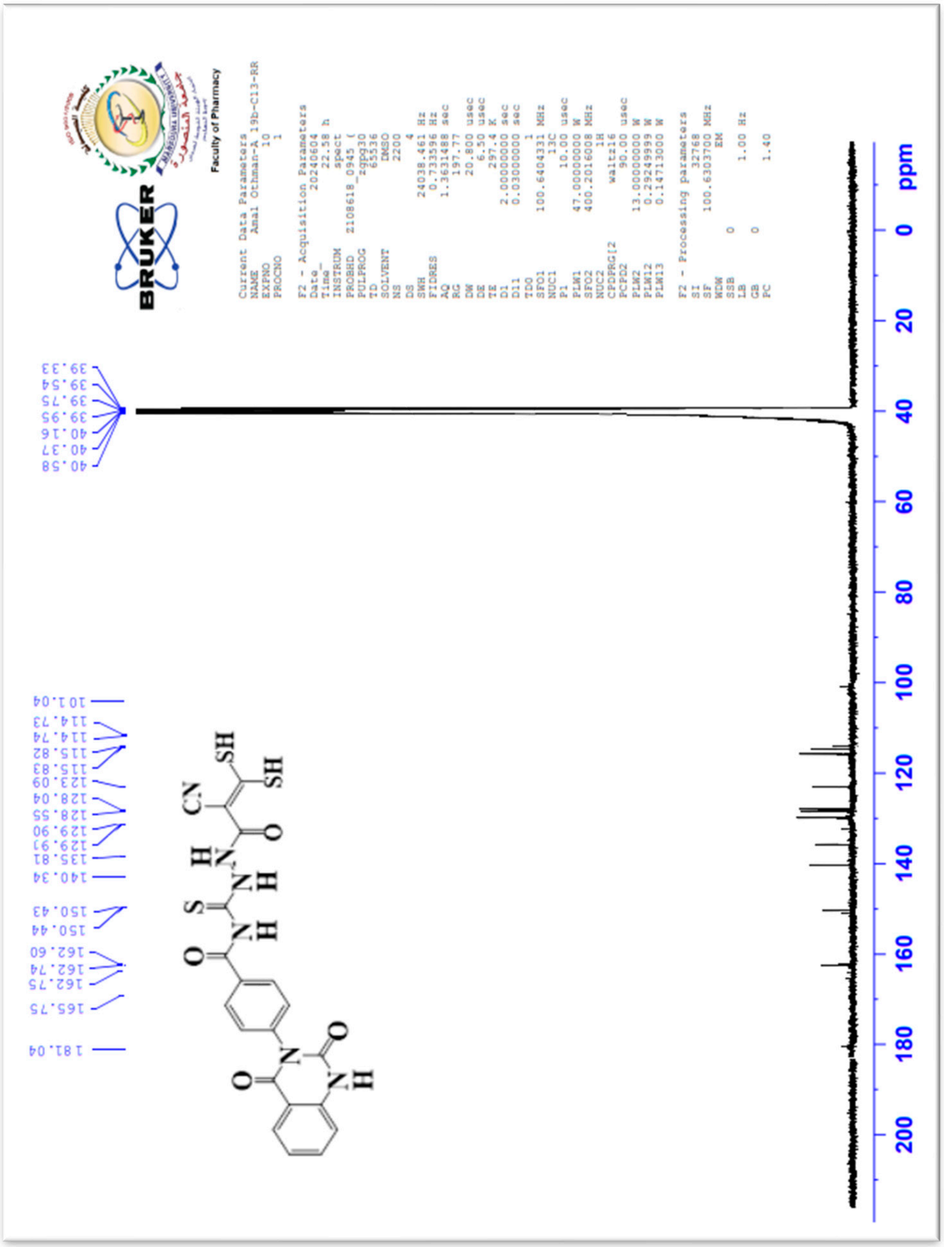

Figure S31: ^{13}C-NMR spectrum of compound 3d

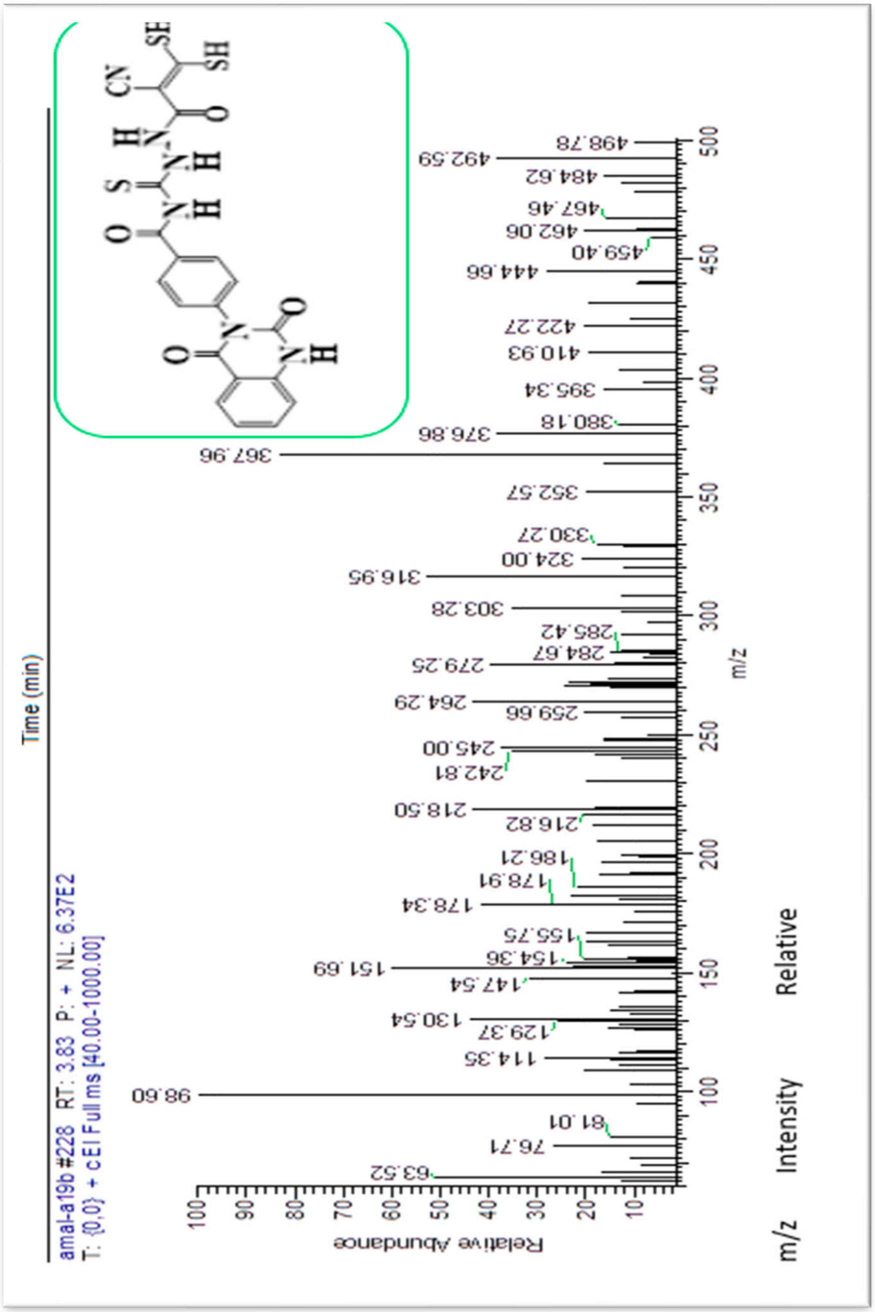

Figure S32: Mass spectrum of compound 3d

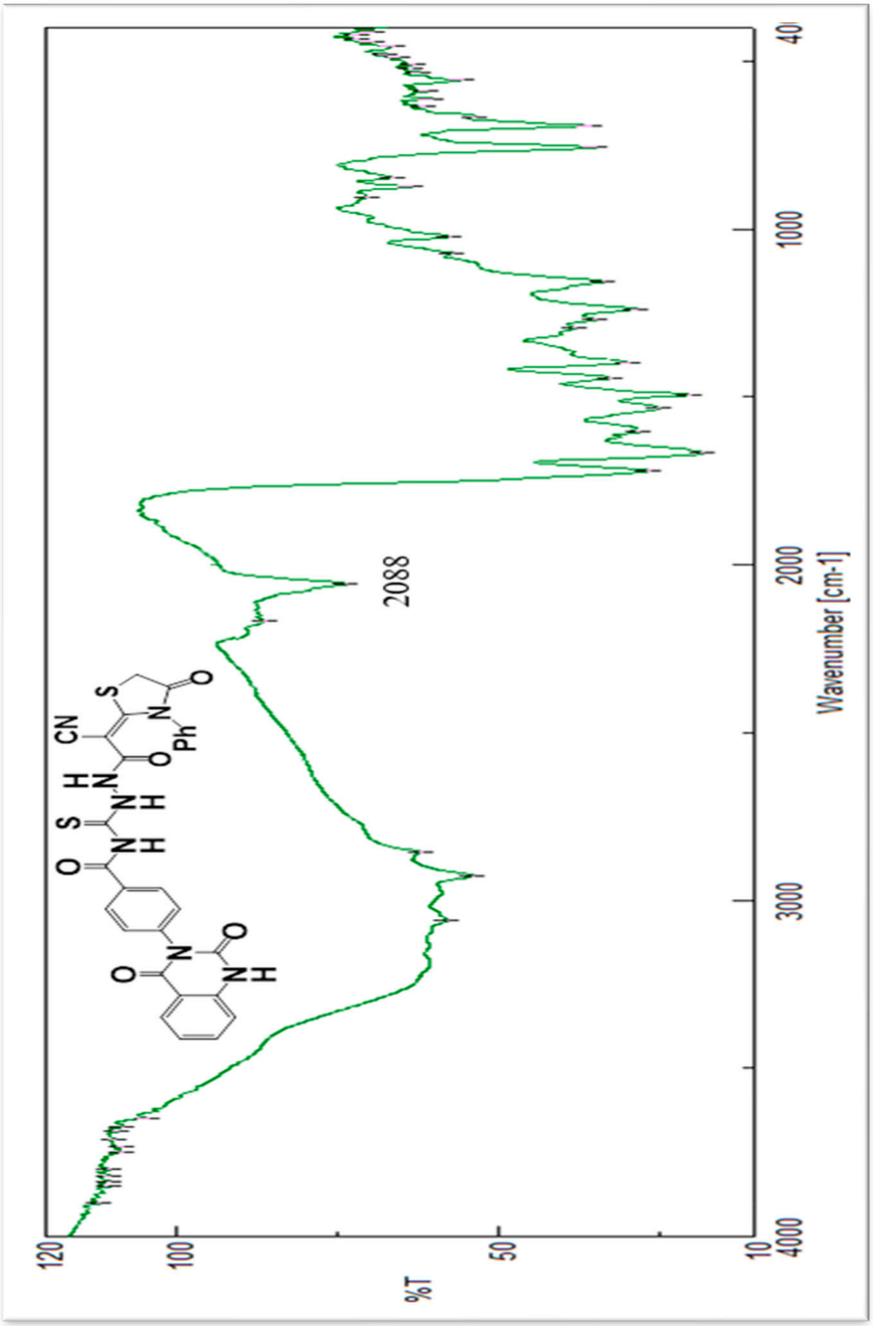

Figure S33: IR spectrum of compound 4a

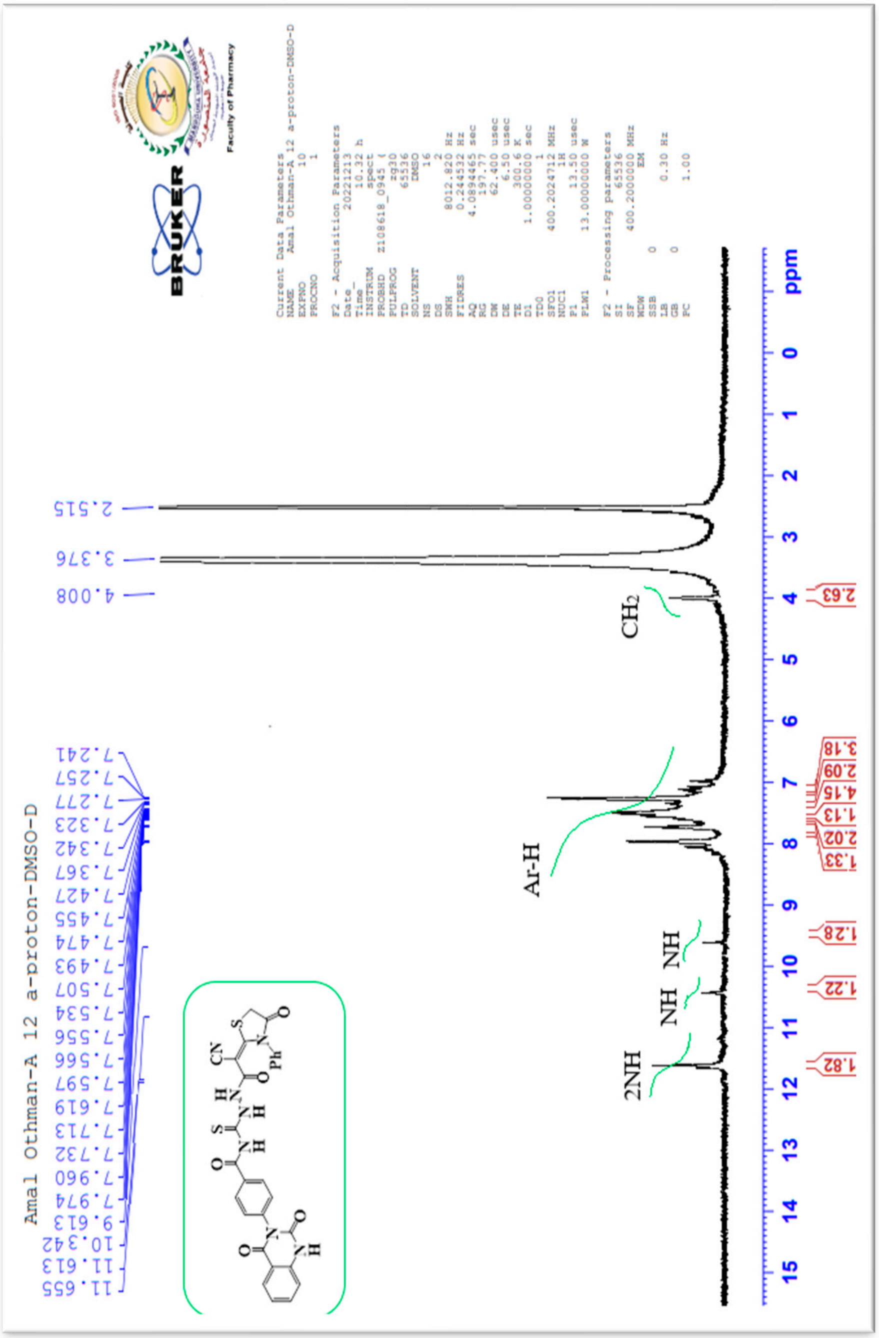

Figure S34: ^{1}H-NMR spectrum of compound 4a

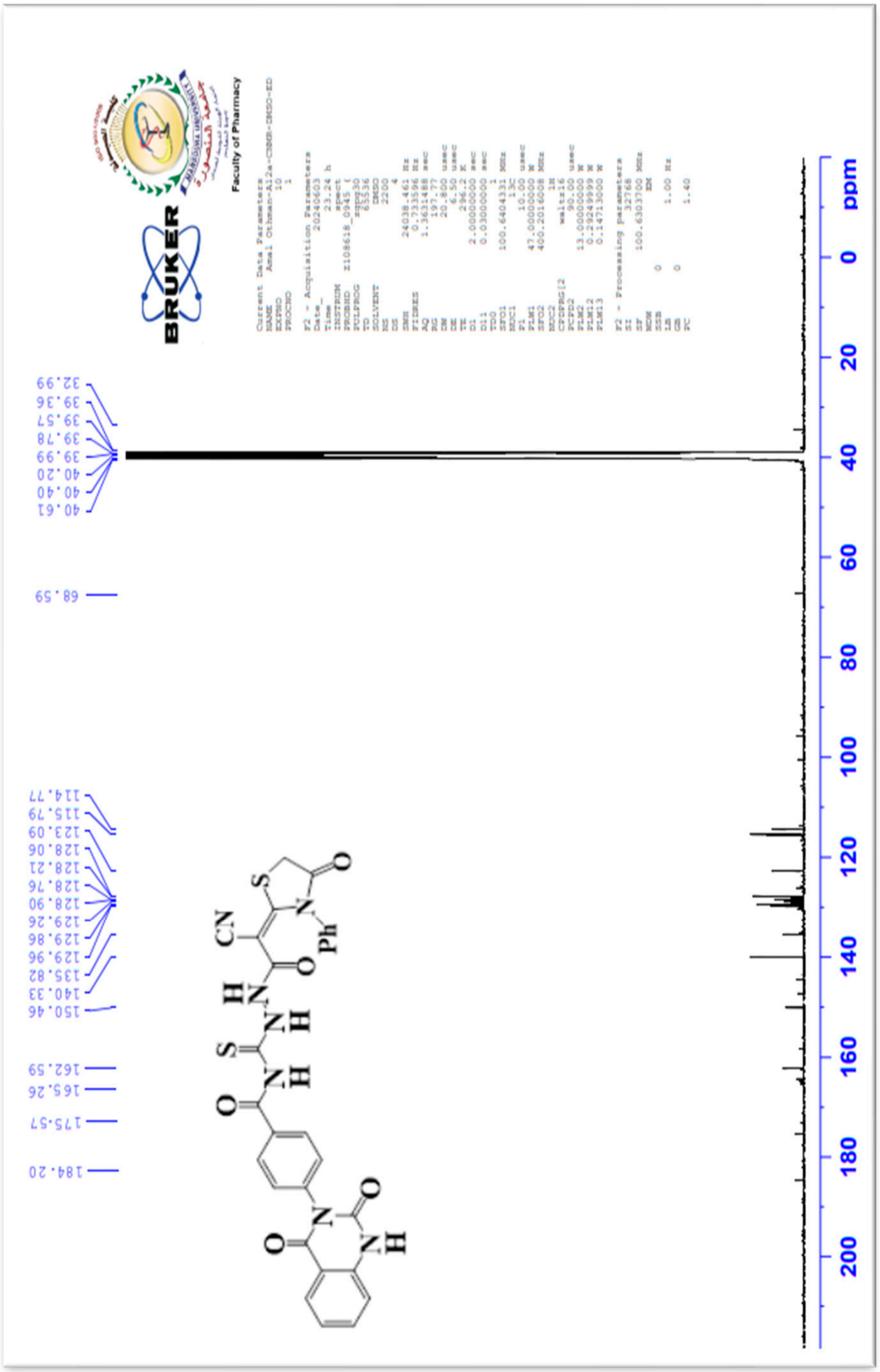

Figure S35: ^{13}C-NMR spectrum of compound 4a

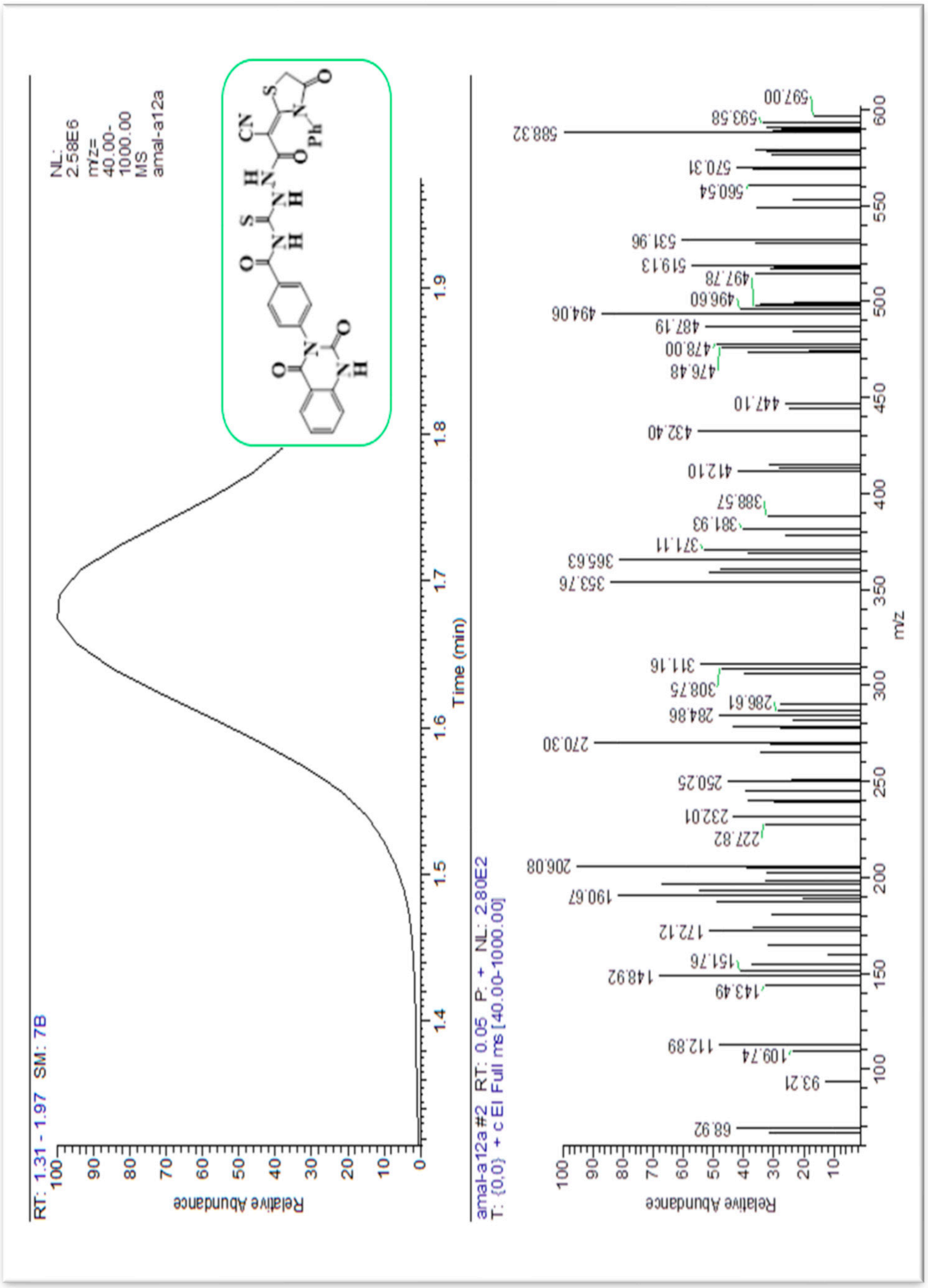

Figure S36: Mass spectrum of compound 4a

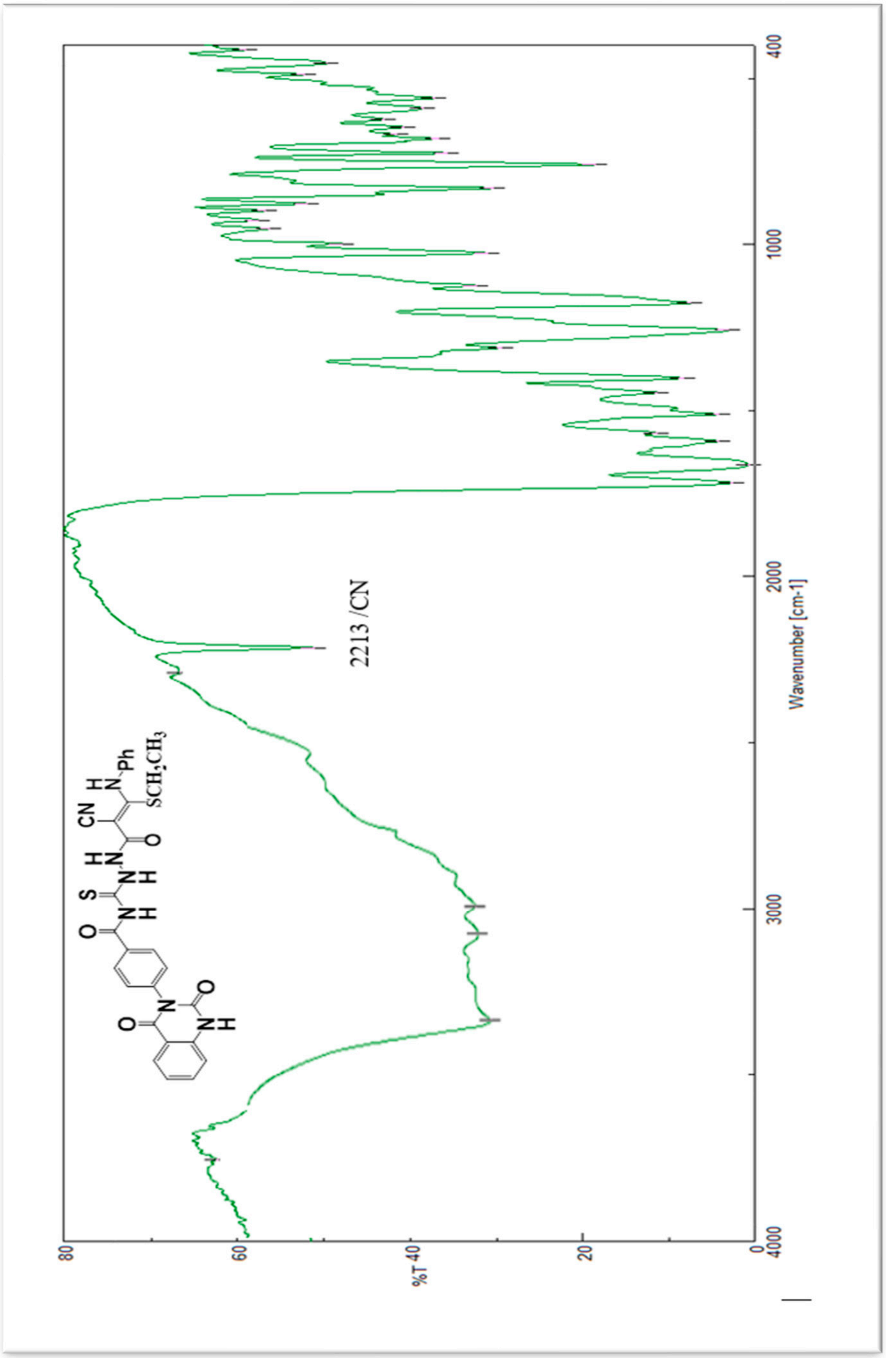

Figure S37: IR spectrum of compound 4b

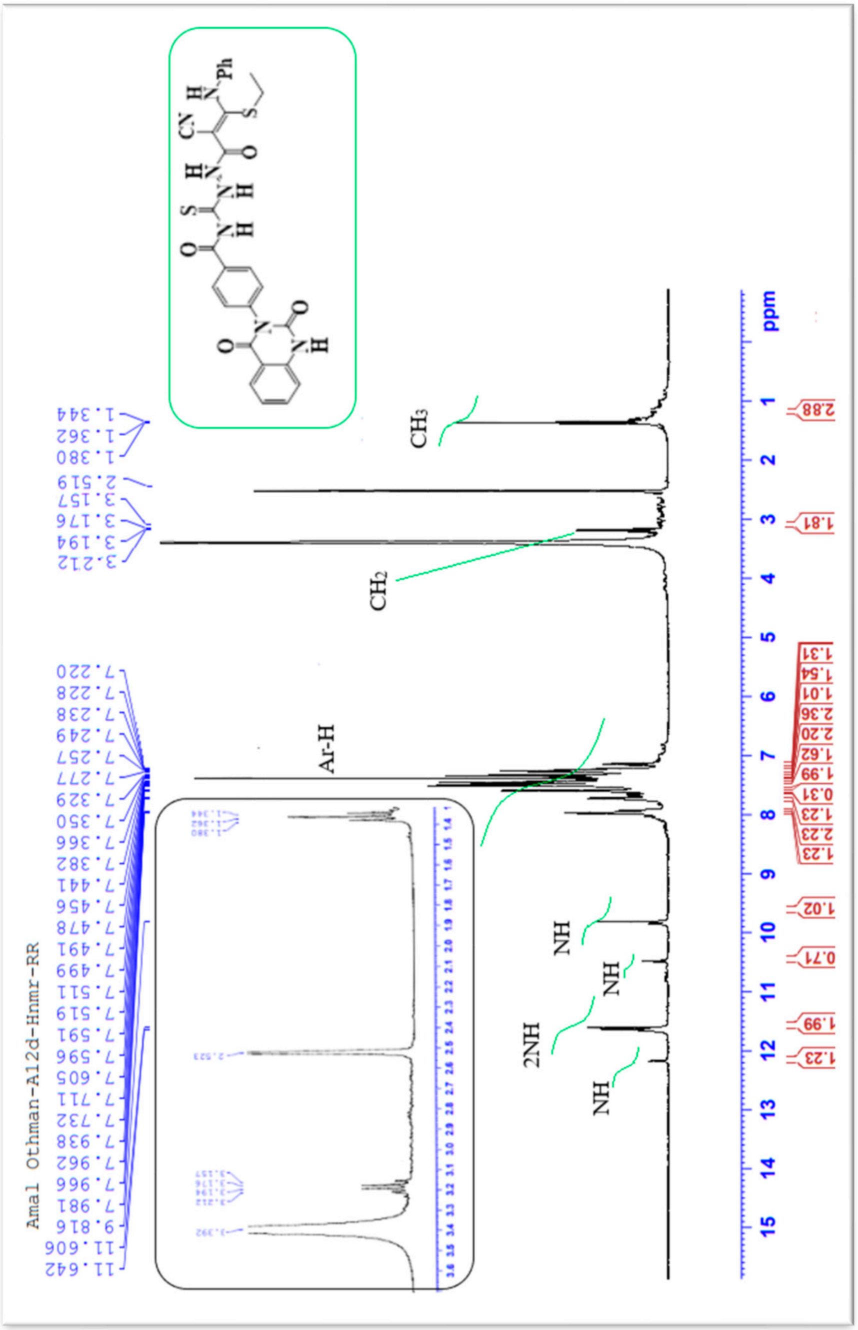

Figure S38: ^{1}H-NMR spectrum of compound 4b

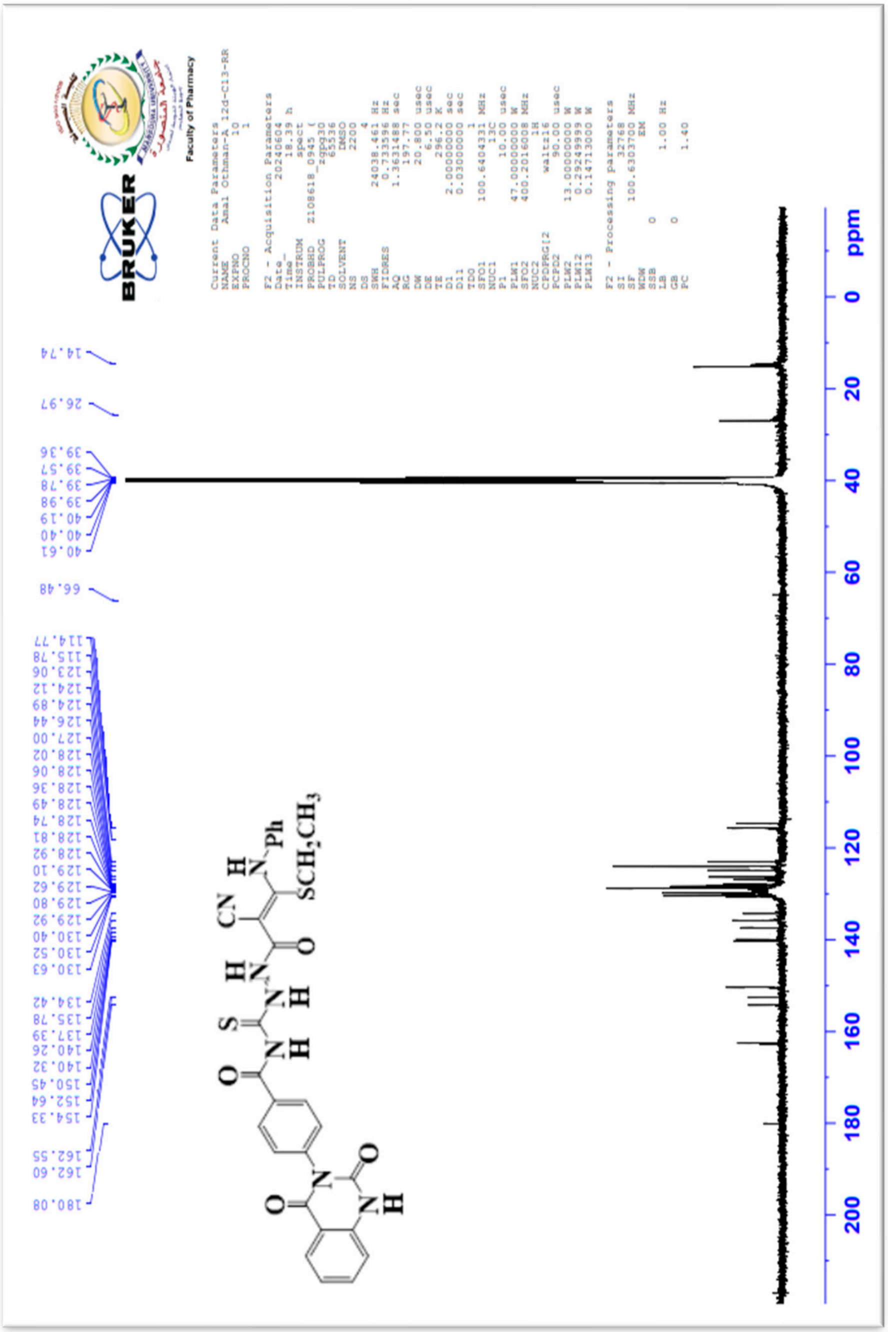

Figure S39: ^{13}C-NMR spectrum of compound 4b

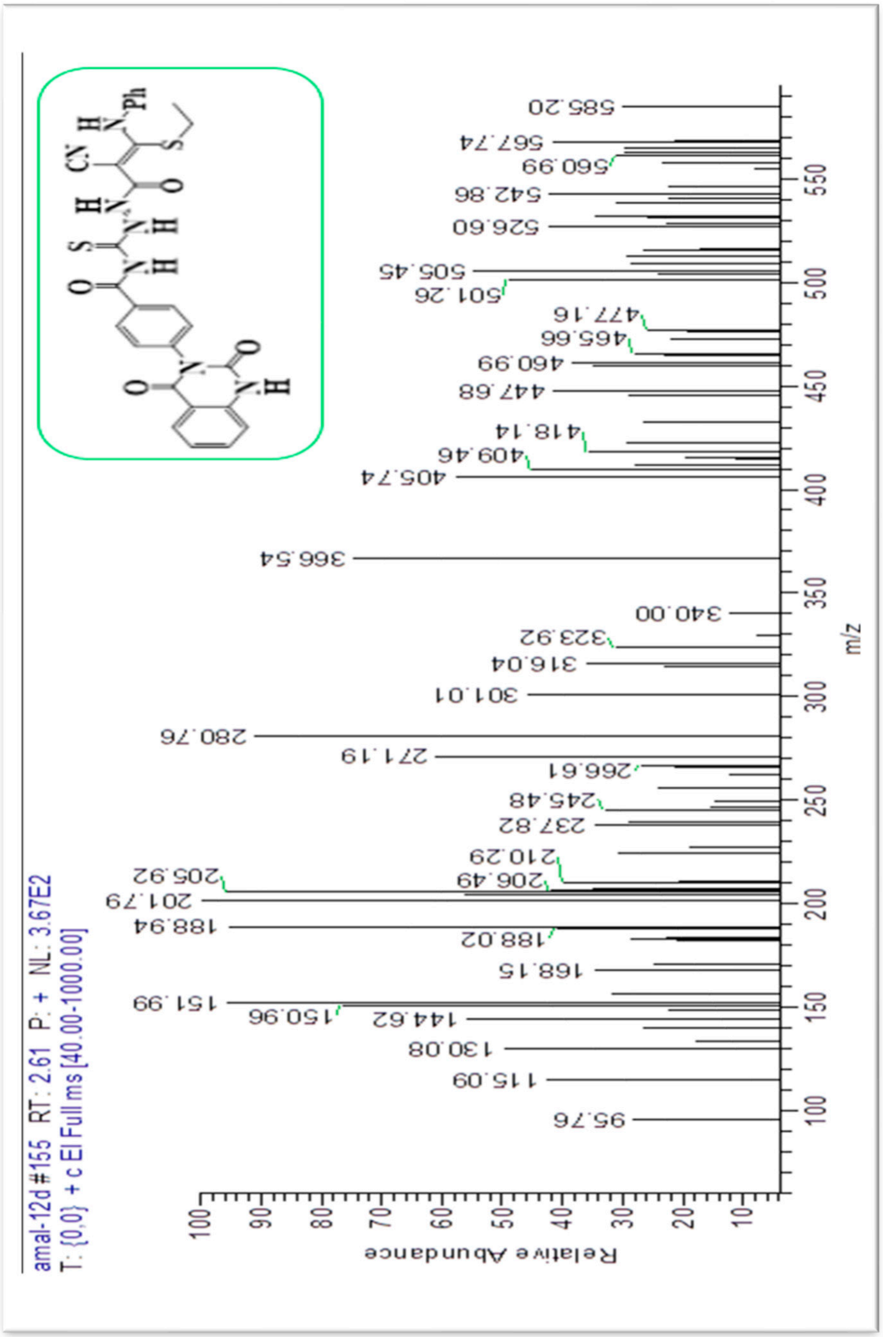

Figure S40: Mass spectrum of compound 4b

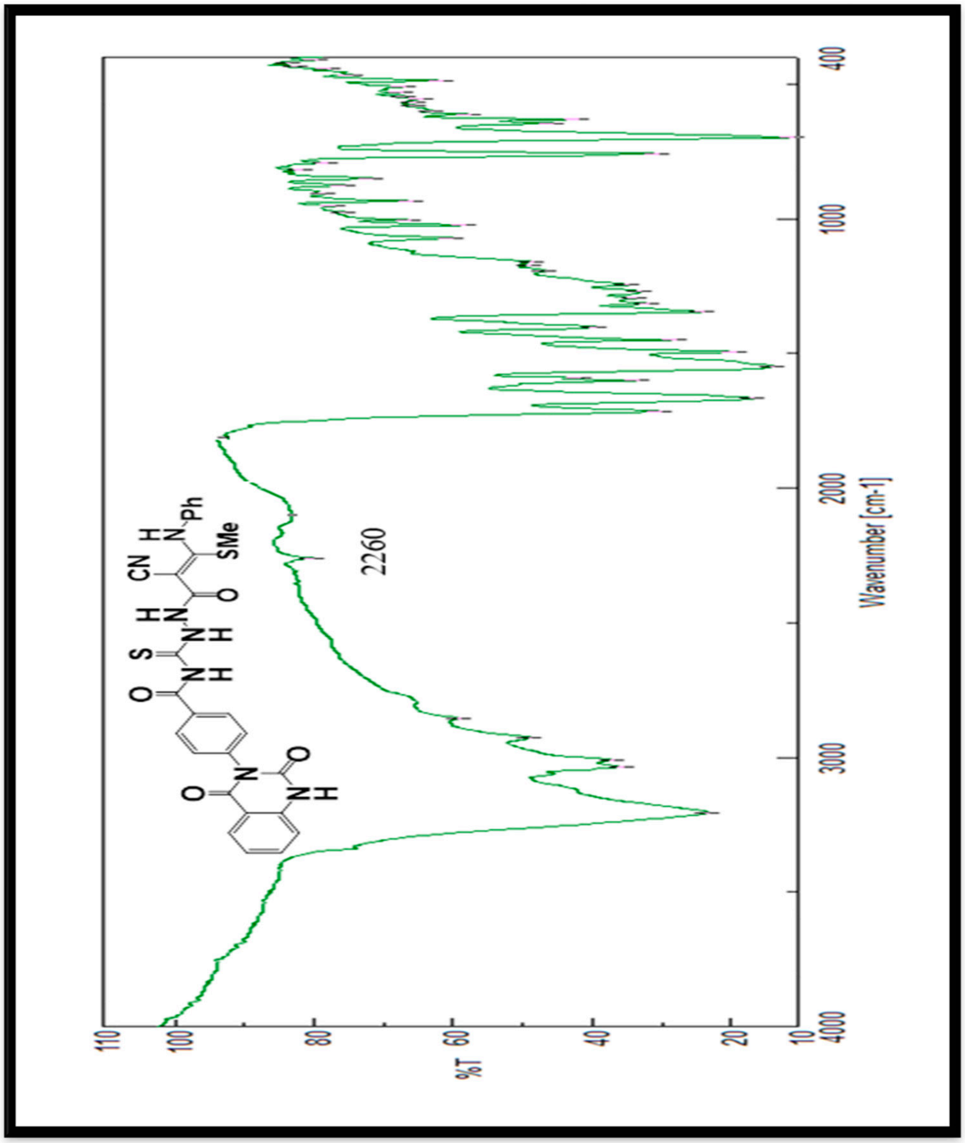

Figure S41. IR spectrum of compound 4c

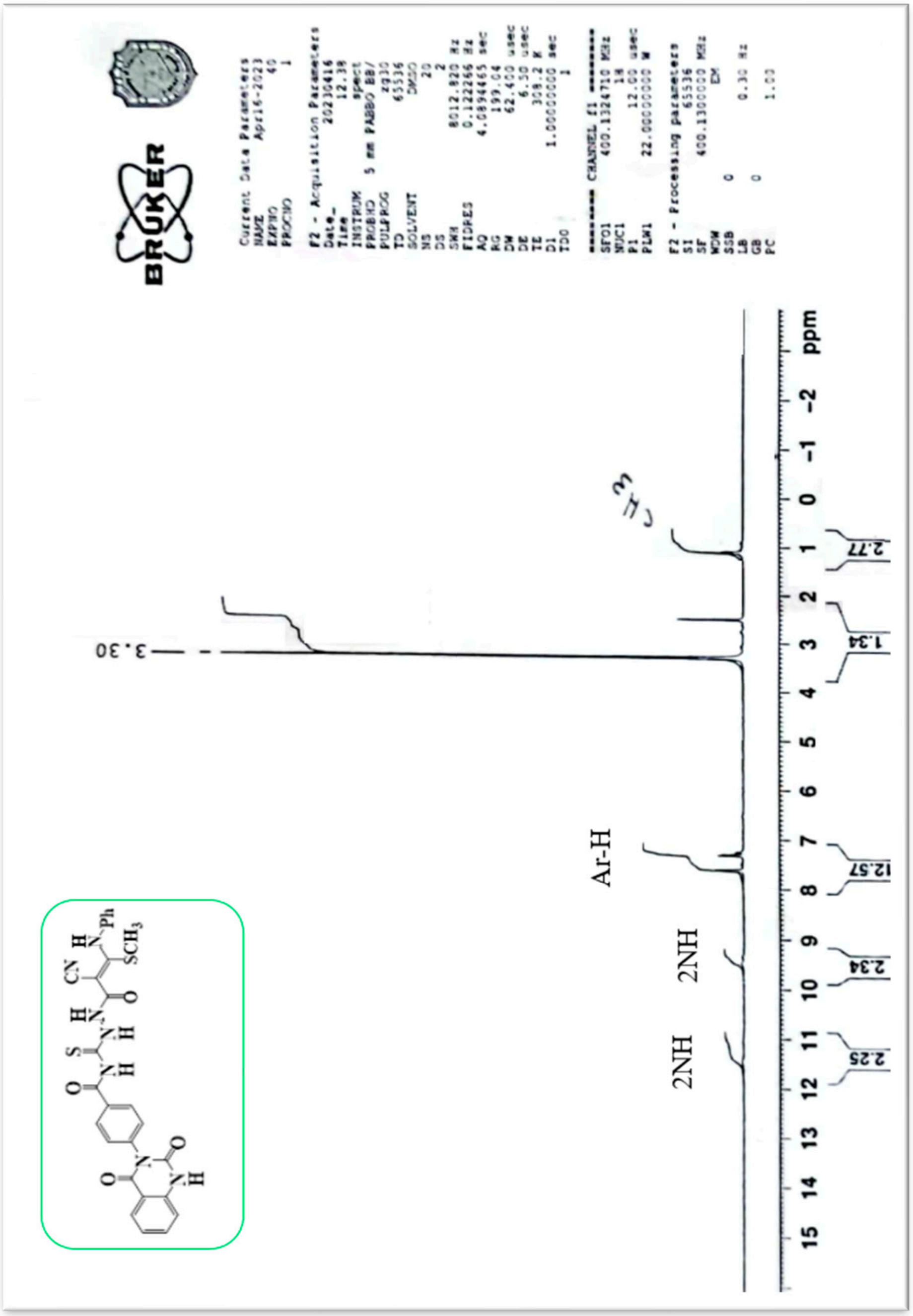

Figure S42: ^{1}H-NMR spectrum of compound 4c

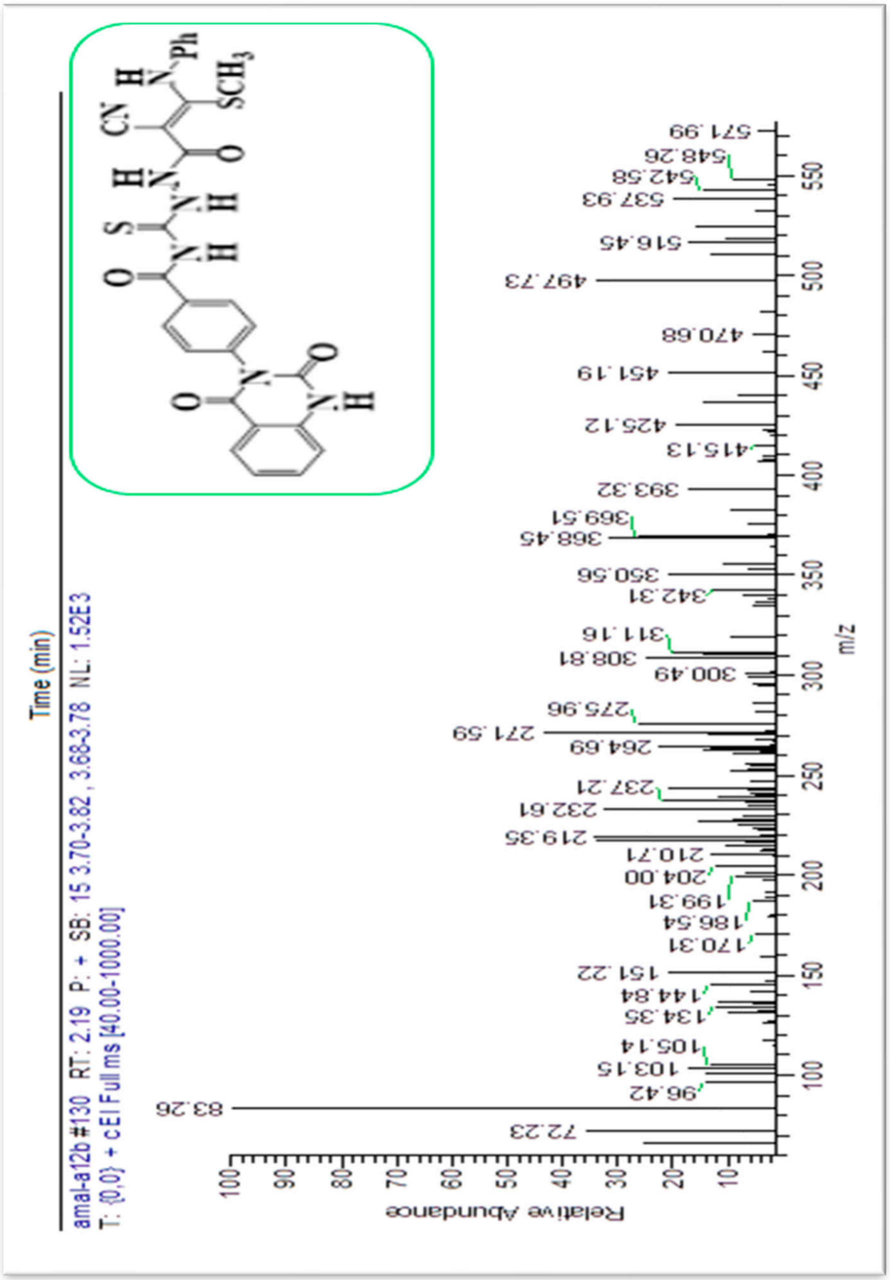

Figure S43: Mass spectrum of compound 4b

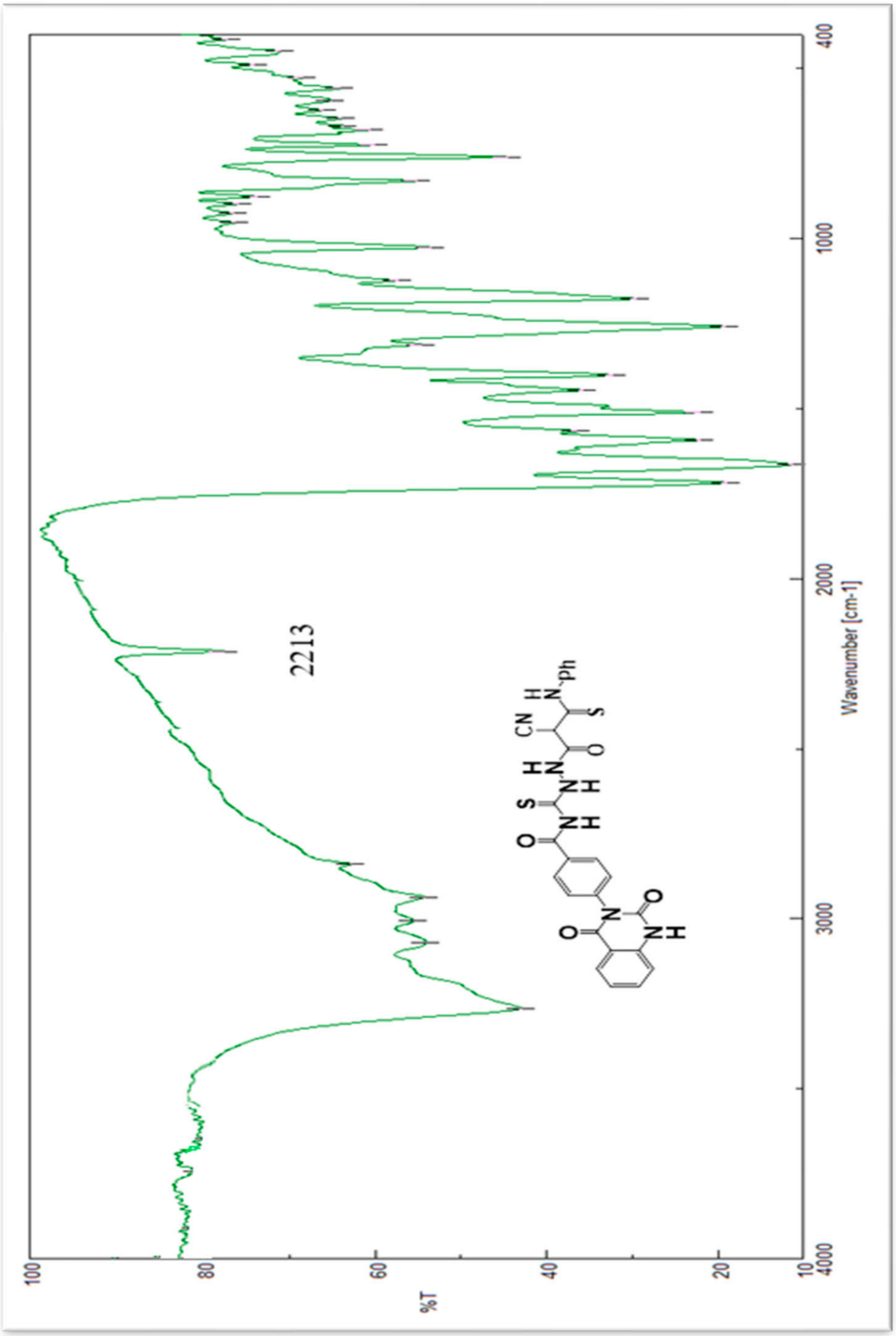

Figure S44: IR spectrum of compound 4d

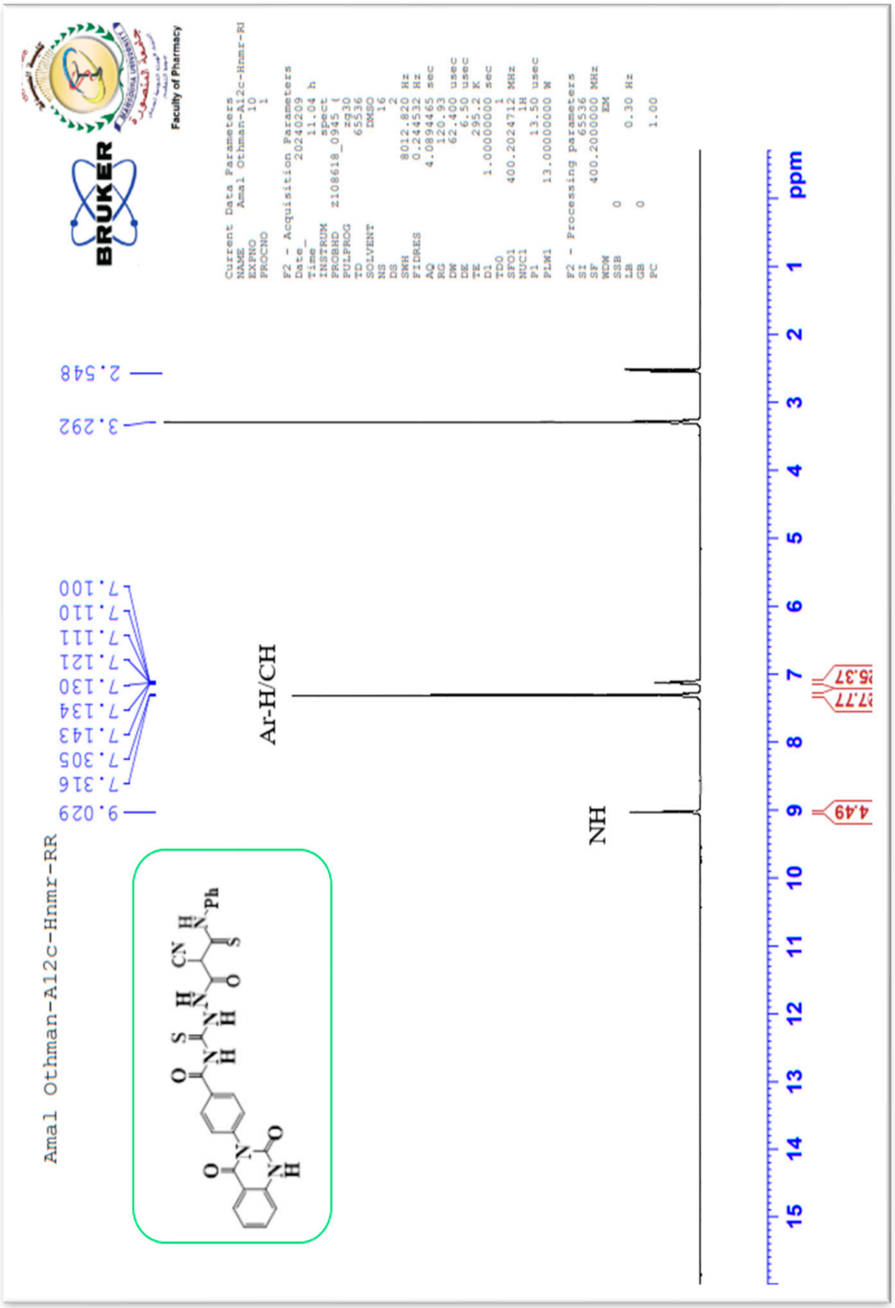

Figure S45: ^{1}H-NMR spectrum of compound 4d

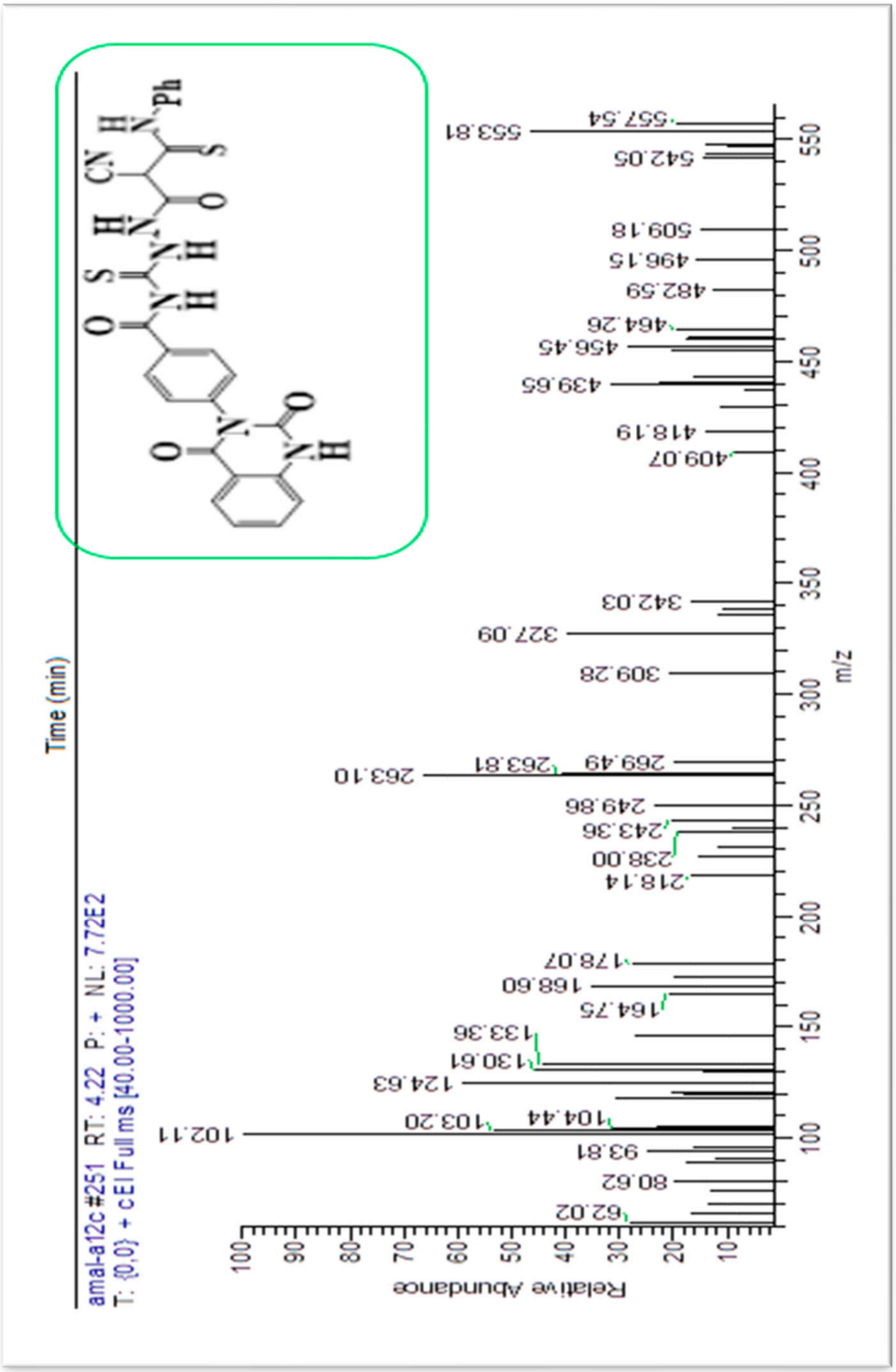

Figure S46: Mass spectrum of compound 4d

**Compound 2a**	**Compound 3a**
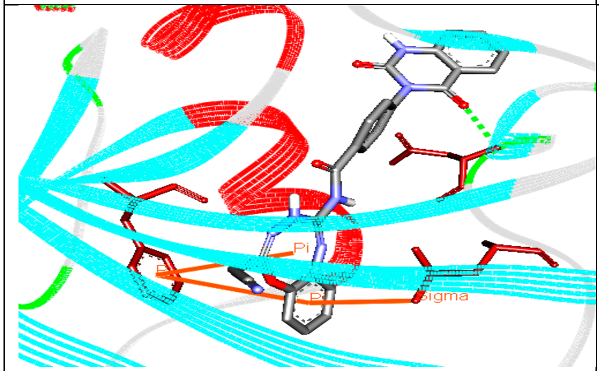	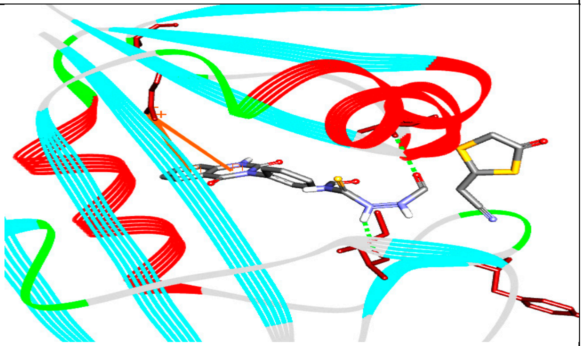
**Compound 3b**	**Compound 3c**
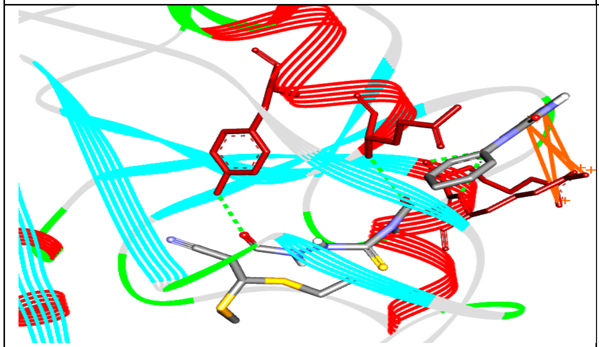	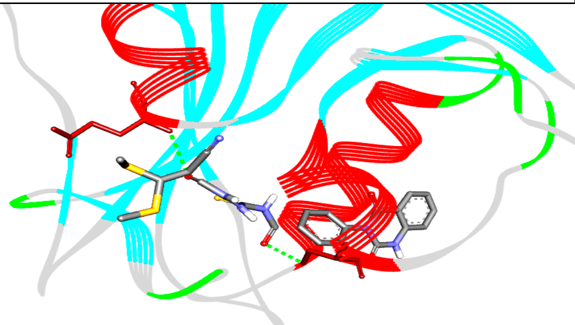
**Compound 3d**	**Compound 4a**
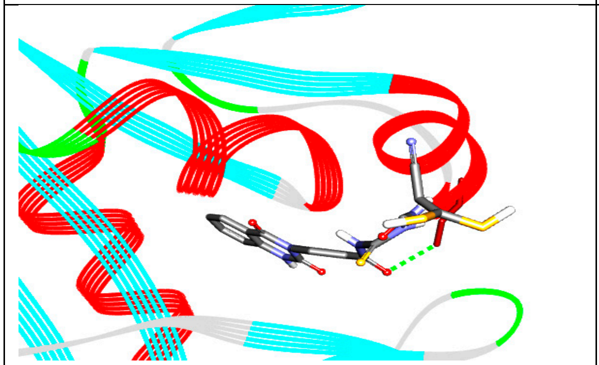	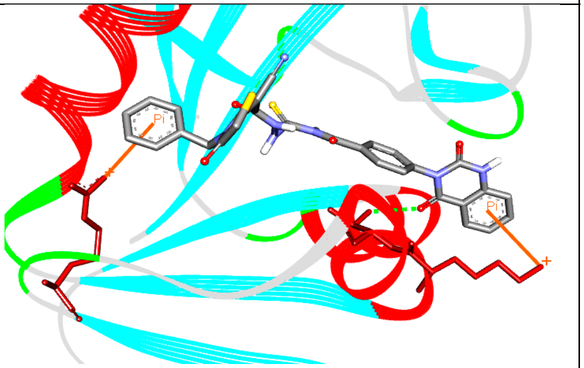
**Compound 4b**	**Compound 4c**

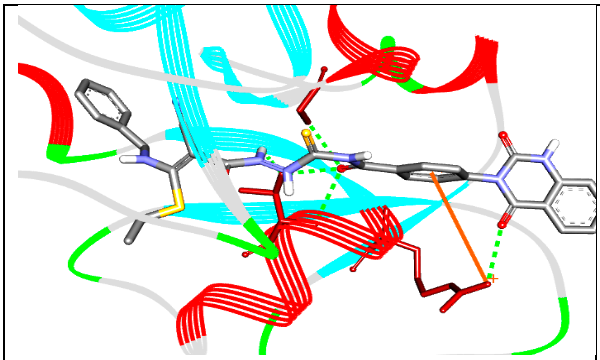	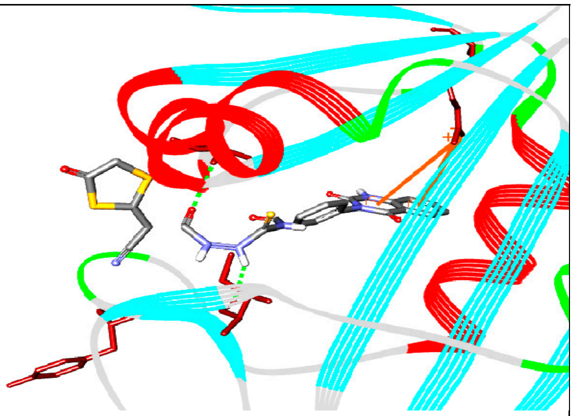
**Compound 4d**	
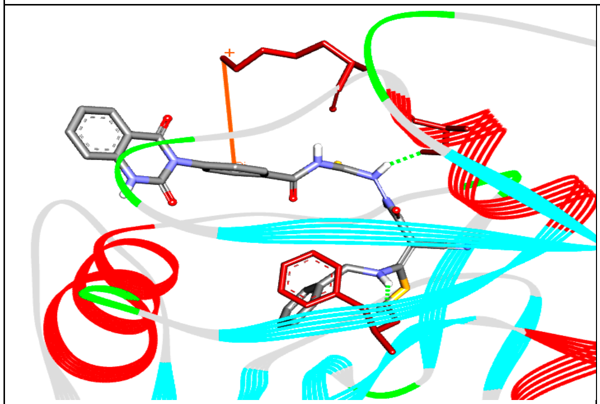	

Figure S47. 3D interactions of the other docked compounds against 2W9S

**Compound 2a**	**Compound 3a**
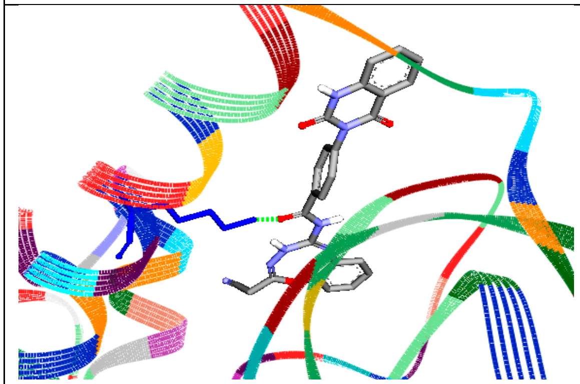	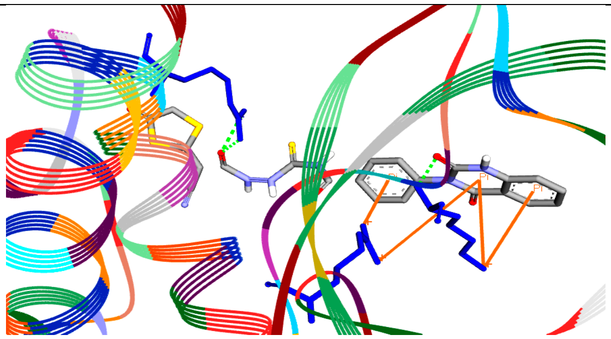
**Compound 3b**	**Compound 3c**

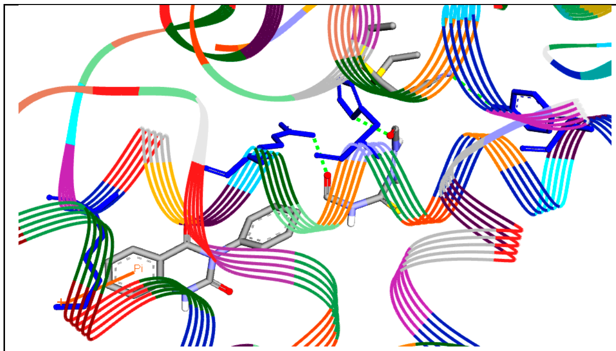	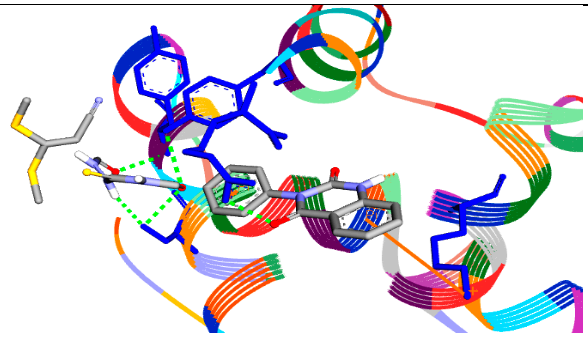
**Compound 3d**	**Compound 4a**
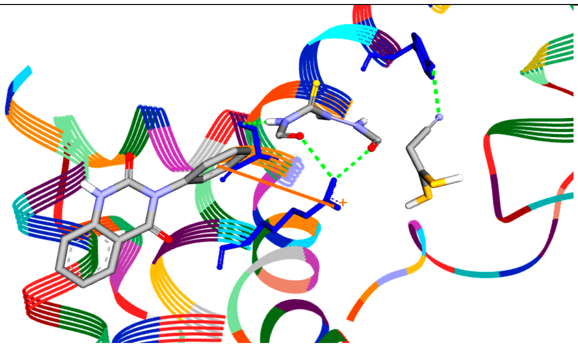	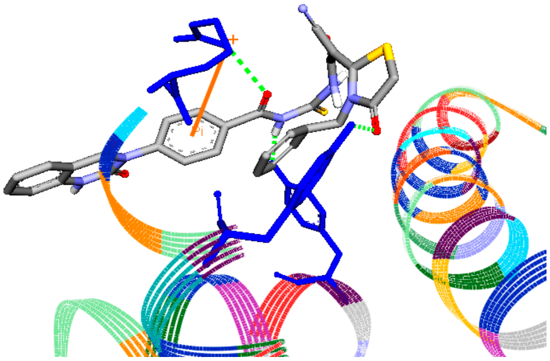
**Compound 4b**	**Compound 4c**
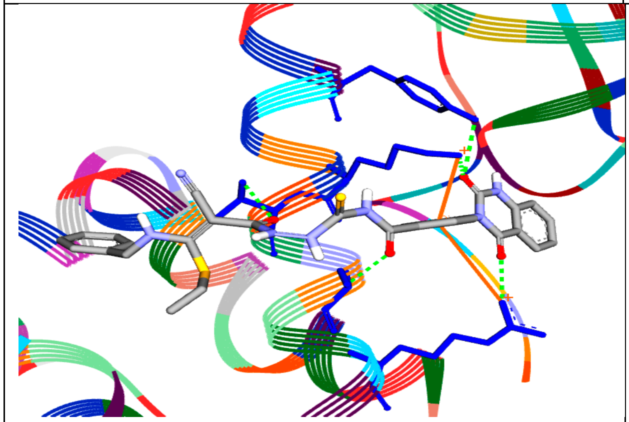	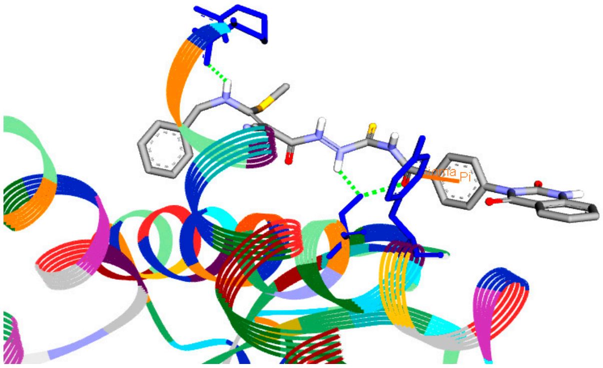
**Compound 4d**	

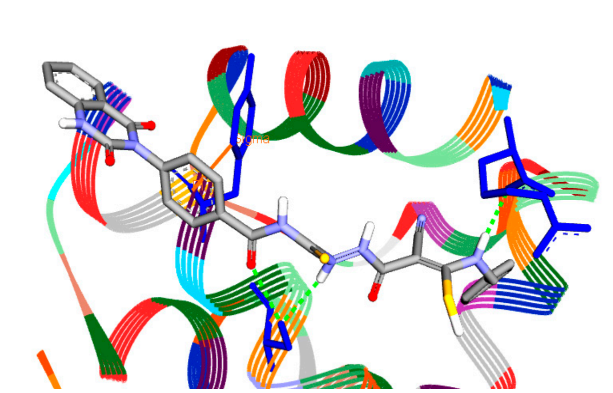

Figure S48. 3D interactions of the other docked compounds against 1Q46
